# State Spending Growth Benchmarks and Hospital Revenue, Hospital Prices, and Premiums

**DOI:** 10.1001/jamanetworkopen.2025.58283

**Published:** 2026-02-20

**Authors:** Christine Eibner, Elizabeth C. Chase, Rose Kerber, Jodi L. Liu

**Affiliations:** 1 RAND Corporation, Arlington, Virginia; 2 RAND Corporation, Boston, Massachusetts; 3 RAND Corporation, Santa Monica, California

## Abstract

**Importance:**

Since 2013, 9 states have implemented health care spending growth benchmarks. States track performance against these benchmarks, and some states can penalize noncompliance. While subsequent growth has frequently exceeded the benchmark, the approach could slow growth relative to what would have happened without benchmark implementation.

**Objective:**

To assess the association between spending growth benchmarks and spending outcomes.

**Design, Setting, and Participants:**

This population-based case-control study used event study models that compare hospitals and counties in states with benchmarks with entropy-balanced comparators in nonimplementing states from January 1, 2015, to December 31, 2025.

**Exposures:**

Implementation of state spending growth benchmarks in Vermont (2018), Delaware (2019), Rhode Island (2019), Connecticut (2021), Oregon (2021), Washington (2022), New Jersey (2023), and California (2025). Massachusetts (2013) was excluded.

**Main Outcomes and Measures:**

Outcomes of interest included hospital inpatient net revenue per discharge and outpatient net revenue per discharge equivalent from the Centers for Medicare & Medicaid Services Health Care Cost Report Information System, mean county-level inpatient and outpatient standardized prices from the RAND Hospital Price Transparency data, and county-level individual and small group market single bronze premiums from the Center for Consumer Information and Insurance Oversight.

**Results:**

A total of 298 hospitals and 184 counties in 8 states that implemented benchmarks and 4515 hospitals and 2924 counties in nonimplementing states were included, yielding a final sample of 4813 hospitals and 3108 counties. On average, no statistically significant changes from preimplementation to postimplementation relative to comparators were found for hospital inpatient revenue (−$839 [95% CI, −$4276 to $1874]), hospital outpatient revenue ($439 [95% CI, −$1207 to $2126]), inpatient prices (−$3 [95% CI, −$2290 to $2417]), outpatient prices ($11 [95% CI, −$12 to $34]), individual market premiums ($8 [95% CI, −$8 to $26]), or small group premiums ($11 [95% CI, −$3 to $25]). State-specific findings were similar, except for Rhode Island, for which benchmarks were associated with a statistically significant $55 (95% CI, −$88 to −$20) reduction in outpatient prices.

**Conclusions and Relevance:**

In this case-control study of hospital revenue, hospital prices, and premiums, scant evidence suggested that spending growth benchmarks were associated with reductions in the outcomes of interest. States may need to pair benchmarks with more rigorous enforcement or additional policies to meaningfully influence health care spending.

## Key Points

**Question:**

Are state health care spending growth benchmarks associated with hospital revenue, hospital prices, or marketplace premiums?

**Findings:**

In this case-control study of 4813 hospitals and 3108 counties that used entropy-balanced difference-in-differences models with 5 to 7 years of postintervention data in 8 intervention states, there was no evidence that spending growth benchmarks were associated with reduced hospital revenue or lower prices. Premium results were mixed, with no evidence of sustained reductions over time.

**Meaning:**

These findings suggest that health care spending growth benchmarks may not be an effective cost-containment mechanism unless paired with stronger enforcement or other reforms.

## Teaser

This case-control study evaluates whether the implementation of health care spending growth benchmarks is associated with reductions in net hospital revenue, hospital prices, or individual or small group premiums.

## Introduction

State health care spending growth benchmarks, which seek to constrain future health spending growth within a percentage of current levels, are a burgeoning strategy to reduce health care costs. The approach was first implemented in Massachusetts, which since 2013 has aimed to keep per capita spending growth under a predetermined limit, ranging from 3.1% to 3.6%. Since then, 8 additional states have adopted benchmarks, with spending growth goals in comparable ranges.

Despite their increasing popularity, the evidence supporting these programs is limited. Based on states’ annual reports, most states that have implemented benchmarks have stayed within their target growth rates fewer than half of the years that programs have been in effect (eTable 1 in Supplement 1). Some states, including Connecticut and Oregon, have never stayed within their benchmark growth rates. However, state annual reports on benchmarks’ performance lack a comparison group, making it difficult to know whether the benchmarks reduced spending relative to what would have occurred in their absence. The Massachusetts Health Policy Commission has argued that, despite not always meeting the benchmark, health care spending in Massachusetts slowed relative to other states after implementation.^1^

In this report, we used a difference-in-differences (DD) methodology to estimate whether the implementation of such benchmarks was associated with reductions in net hospital revenue, hospital prices, or individual or small group premiums. Our approach compared hospital revenue, hospital prices, and premiums in areas that were affected by spending growth benchmarks with those in areas that were not affected. By using a DD approach, we were able to address the possibility that the benchmarks slowed growth, regardless of whether states met their benchmarks. We also looked across states and over time to determine whether associations were stronger in states with enforcement policies and to ascertain whether associations changed as the programs matured.

## Methods

This case-control study was approved by the Institutional Review Board of RAND, which waived the need for informed consent because the study was deemed non–human participant research. The study was preregistered at Open Science and followed the Strengthening the Reporting of Observational Studies in Epidemiology (STROBE) reporting guideline.

### Study Design

To measure hospital revenue, we used the RAND Hospital Data (RHD), which are derived from the Center for Medicare & Medicaid Services (CMS) Health Care Cost Reporting Information System (HCRIS). All Medicare-certified hospitals are required to submit comprehensive cost information through HCRIS on an annual basis, and thus the RHD contain comprehensive discharge data from all hospitals that receive Medicare reimbursement. We used these data to measure hospital net revenue per inpatient discharge and per outpatient discharge equivalent, variables that have been used in prior work as proxies for price.^2,3^ A discharge equivalent expresses the volume of outpatient services in terms of inpatient discharges; the total number of outpatient discharge equivalents represents the number of inpatient discharges that could be delivered with the same level of outpatient operating expenses.^4^ To avoid bias from hospitals with few discharges, we limited the inpatient sample to hospitals with at least 100 inpatient discharges in all years of analysis; similarly, we required 100 outpatient discharge equivalents in all years of analysis for the outpatient revenue outcome.

To measure price directly, we used the RAND Hospital Price Transparency Data (HPTD), which are compiled by RAND through submissions from self-insured employers and state all-payer claims databases (APCDs) that predominantly contain fully insured plans and public sector plans.^5^ We used county-level mean prices as our outcome of interest. We excluded counties with fewer than 11 claims in any year to avoid bias from small sample sizes. We standardized both inpatient and outpatient prices to reflect service intensity and case mix, which avoids attributing higher prices to hospitals that provide complex services.^6^ Although the HPTD represent only about 6% of hospital claims nationally,^6^ for treatment states other than Washington, contributors are primarily APCDs and the prices are dominated by fully insured contributors. Although Washington has an APCD, it was not available for this analysis. To address these issues, we included a time-varying control for the share of claims that came from a state’s APCD in our regressions.

Last, we measured individual and small group premiums using data reported by the CMS Center for Consumer Information and Insurance Oversight and compiled by the Robert Wood Johnson Foundation. For both the individual and small group markets, we analyzed the mean bronze or expanded bronze premiums for a nonsmoking patient aged 27 years. Vermont, a treatment state, has full community rating, which makes their premiums for patients aged 27 years much higher than in other states. Further, Vermont unmerged its individual and small group markets in 2022,^7^ which affected premium trends in the postintervention period. Given these issues, we excluded Vermont from the premium analysis. More information on the construction of the hospital and county samples is given in eFigures 1 and 2 and eTables 2 and 3 in Supplement 1.

Table 1 provides descriptive information on the outcomes of interest and data considered in our analysis. For all analyses, our comparison group consists of hospitals or counties in states that did not have a benchmark in any of the posttreatment years available for the outcome. We excluded Massachusetts and Maryland due to significant overlapping reforms (hospital rate setting in Maryland and the health insurance reforms of Chapter 58 in Massachusetts) that make these states difficult to compare with other states. Some of the remaining implementor states could not be included in all analyses because the latest year of available data predated implementation of the benchmark—this affected California in the RHD and both California and New Jersey in the HPTD. In these cases, we used these states as untreated controls. eTable 4 in Supplement 1 provides a complete listing of the treated and control states for each outcome. All dollar values are analyzed in nominal terms.

**Table 1.** Hospital and Insurance Outcomes of Interest

Variable	Outcome					
	Inpatient revenue per discharge	Outpatient revenue per discharge equivalent	Standardized inpatient prices	Standardized outpatient prices	Individual single bronze premium	Small group single bronze premium
Source	RHD	RHD	HPTD	HPTD	CCIIO RWJF	CCIIO RWJF
Unit of observation	Hospital	Hospital	County	County	County	County
Years in analysis by state (target effective date)						
Vermont (2018)	2015-2023	2015-2023	2016-2022	2016-2022	2015-2025	2015-2024
Delaware (2019)	2016-2023	2016-2023	2016-2022	2016-2022	2016-2025	2016-2024
Rhode Island (2019)	2016-2023	2016-2023	2016-2022	2016-2022	2016-2025	2016-2024
Connecticut (2021)	2018-2023	2018-2023	2018-2022	2018-2022	2018-2025	2018-2024
Oregon (2021)	2018-2023	2018-2023	2018-2022	2018-2022	2018-2025	2018-2024
Washington (2022)	2019-2023	2019-2023	2019-2022	2019-2022	2019-2025	2019-2024
New Jersey (2023)	2020-2023	2020-2023	NA	NA	2020-2025	2020-2024
California (2025)	NA	NA	NA	NA	2022-2025	NA
No. of treated units	294	262	71	85	184	126
Value in treatment states across preintervention years, mean (SD)	$17 578 ($10 657)	$31 106 ($31 469)	$22 328 ($6179)	$324 ($110)	$341 ($59)	$305 ($70)
Value in treatment states across postintervention years, mean (SD)	$18 942 ($13 572)	$33 147 ($29 389)	$24 348 ($6832)	$399 ($110)	$398 ($122)	$382 ($114)
No. of comparison units	4339	3776	794	1302	2924	2982
Value in comparison states across all years, mean (SD)	$14 226 ($10 649)	$26 642 ($22 684)	$22 521 ($13 189)	$398 ($167)	$338 ($83)	$333 ($92)

Abbreviations: CCIIO, Center for Consumer Information and Insurance Oversight; HPTD, Hospital Price Transparency Data; RHD, RAND Hospital Data; RWJF, Robert Wood Johnson Foundation.

### Statistical Analysis

To estimate the association between health care spending targets and the outcomes of interest, we used entropy-balanced DD models.^8^ To address the staggering of benchmarks across state adoption, we conducted separate analyses for each treated state and then aggregated the results, using the set of never-treated states as a constant set of comparators. After conducting the analysis on each treated state, we took the weighted mean of the estimates as our overall estimate, weighting according to the proportion of all treated counties (or hospitals) that came from each treated state.^9,10^

We used entropy balancing (EB) to increase similarity between comparison counties and hospitals and treated counties and hospitals.^11^ We calculated EB weights separately for each outcome and for each treated state. Our first priority with the EB was to minimize differences in the preimplementation trends of the outcome variables, to bolster the parallel trends assumption.^8,12^ For each outcome, we included 2 preimplementation trend measurements in the EB. We also included the following covariates: county population, rural-urban status, whether or not the county was in a Medicaid expansion state, hospital market concentration, the percentage of the population aged 18 to 64 years with health insurance, and the median population age. For the HCRIS outcomes we also included the number of hospital beds and whether it was a critical access hospital, a government-owned hospital, or a short-term general hospital. All of these covariates were measured in the year prior to intervention for that state and were largely derived from Area Health Resource Files^13^ and US Census Bureau data.^14^ eMethods 1 in Supplement 1 provides more details. We balanced on the first and second moments of each variable.^15^ For the individual market premiums outcome we were unable to balance on rural-urban status and the percentage of the population aged 18 to 64 years with health insurance while maintaining adequate effective sample size in the control group, and similarly we were unable to balance on the number of hospital beds for the outpatient HCRIS outcome. We removed these covariates from the EB for these outcomes. More information on the performance of the EB is given in eTables 5 and 6 in Supplement 1.

After estimating the EB weights, we fit weighted DD models that included a treatment indicator, along with state and year fixed effects and time-varying controls.^16^ Time-varying controls included the annual COVID-19 case rate per capita, the annual COVID-19 death rate per capita, and whether Medicaid expansion was in place in that year, along with annual population size, poverty rate, unemployment rate, and percentage of the population 65 years or older. We fit the model separately for each treated state (with a constant set of never-treated control states) and weighted the analysis using both the EB weights and population size (county population for the county-level outcomes, number of hospital beds for the hospital-level outcomes). We also fit an event-study model to estimate how the association changed over time. As with the overall model, we fit the event-study model separately for each treated state, using EB and population weights. We then aggregated the event-study estimates across states by taking the weighted mean with weights proportional to the proportion of treated counties and hospitals in a given year that came from each treated state.^9,10^

We used a fractionated bootstrap to obtain appropriate inference^17^ and multiple imputation to address missing data in our adjuster variables (eMethods 2 and eTable 4 in Supplement 1).^18^ All statistical analyses were conducted in R, version 4.5.0 (R Project for Statistical Computing)^19^; SAS Enterprise Guide, version 8.6.1.13 (SAS Institute Inc) was used to construct the underlying data files. Two-sided *P* < .05 indicated statistical significance. eMethods 3 in Supplement 1 provides additional detail on inference.

## Results

The final sample included 4813 hospitals and 3108 counties. Data were collected from January 1, 2015, to December 31, 2025.

### Hospital Net Revenue Per Discharge

**Figure 1.**
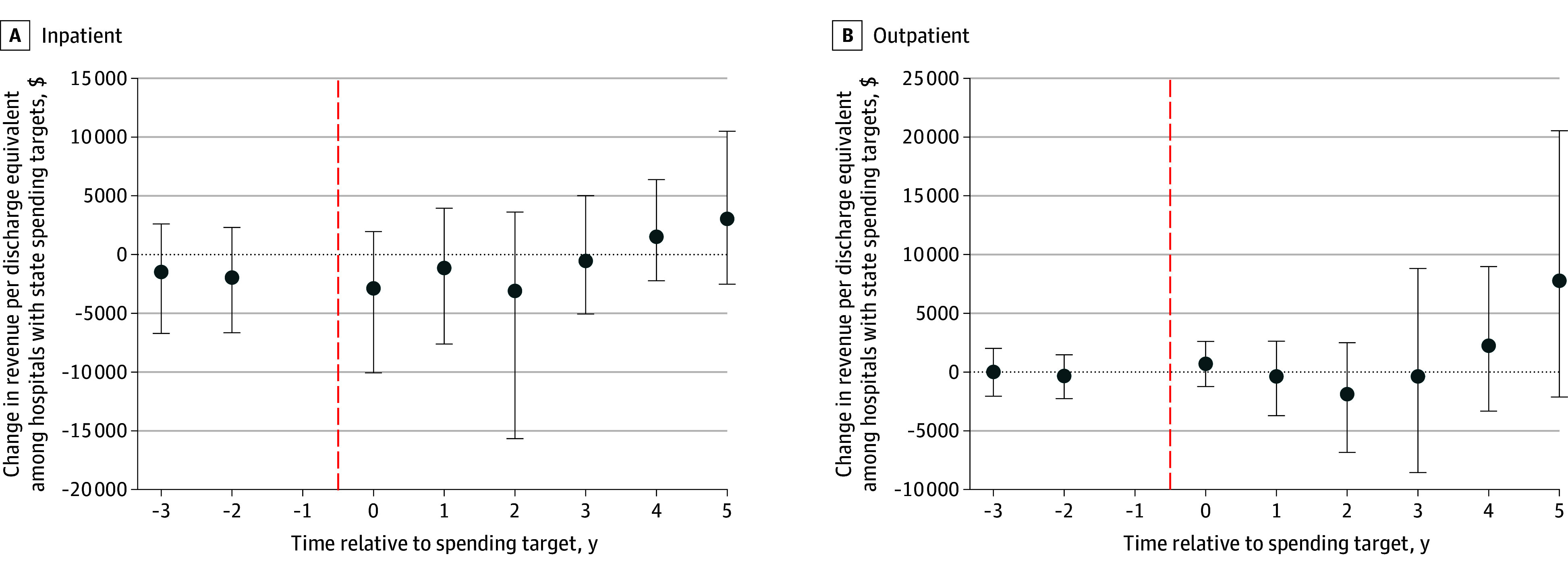
Changes in Inpatient and Outpatient Revenue Per Discharge Equivalent Before and After Benchmark Implementation Relative to Comparators Estimates for later years are based only on the subset of treated states with sufficient follow-up data. Error bars indicate 95% CIs; vertical dashed lines indicate implementation.

Our inpatient sample included 294 treatment and 4339 comparison hospitals, and our outpatient sample included 262 treatment hospitals and 3776 comparators. Figure 1 shows the results of the analysis of benchmark implementation and hospital inpatient revenue per discharge and outpatient revenue per discharge equivalent. Changes ranged from a low of −$3096 (95% CI, −$15 674 to $3621) for the inpatient measure and −$1880 (95% CI, −$6840 to $2505) for the outpatient measure, both in year 2, to a high of $3035 (95% CI, −$2522 to $10 491) for the inpatient measure and $7771 (95% CI, −$2120 to $20 538) for the outpatient measure in year 5. Overall, changes were −$839 (95% CI, −$4276 to $1874) for hospital inpatient revenue and $439 (95% CI, −$1207 to $2126) for hospital outpatient revenue. None of the changes were statistically significant.

### Hospital Prices

**Figure 2.**
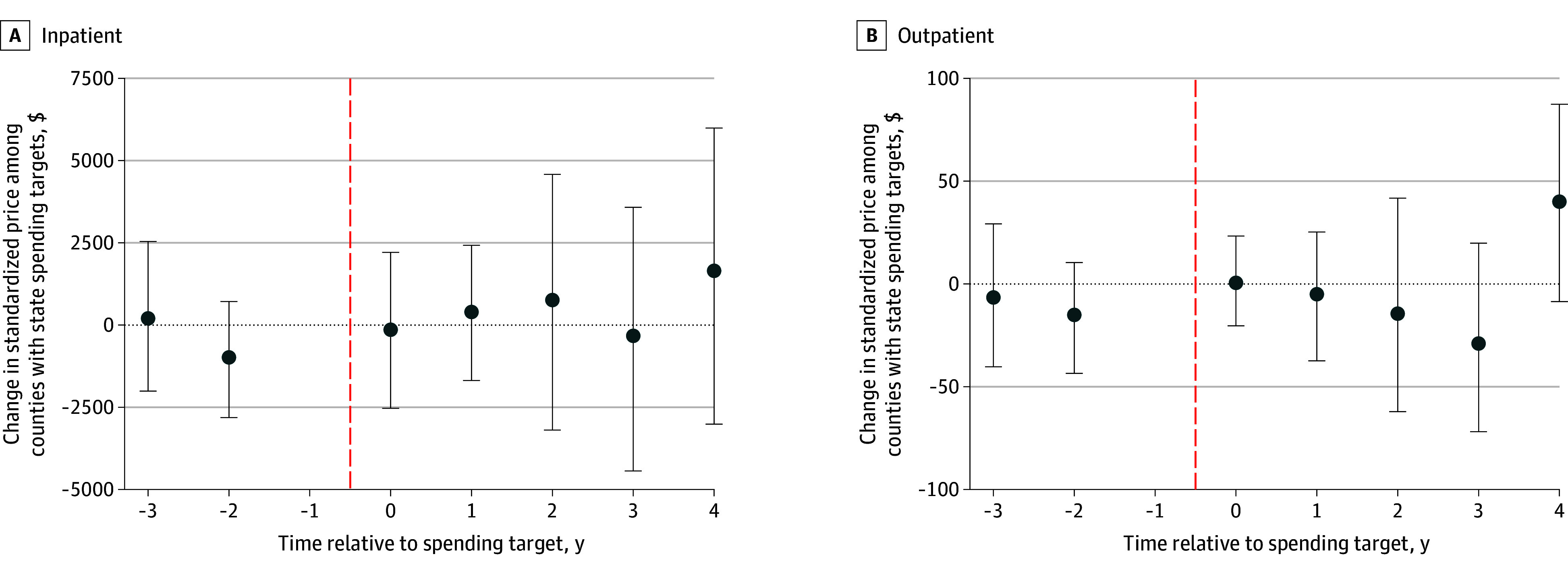
Changes in Standardized Inpatient and Outpatient Prices Before and After Benchmark Implementation Relative to Comparators Estimates for later years are based only on the subset of treated states with sufficient follow-up data. Error bars indicate 95% CIs; vertical dashed lines indicate implementation.

We analyzed standardized inpatient prices in 71 treatment and 794 comparison counties and outpatient prices in 85 treatment and 1302 comparison counties. We found no statistically significant changes (Figure 2). Inpatient price changes ranged from a low of −$328 (95% CI, −$4437 to $3579) in year 3 to a high of $1648 (95% CI, −$3012 to $5985) in year 4 (overall change, −$3 [95% CI, −$2290 to $2417]). Outpatient price changes ranged from −$29 (95% CI, −$72 to $20) in year 3 to $40 (95% CI, −$9 to $87) in year 4 (overall, $11 [95% CI, −$12 to $34]).

### Individual and Small Group Premiums

**Figure 3.**
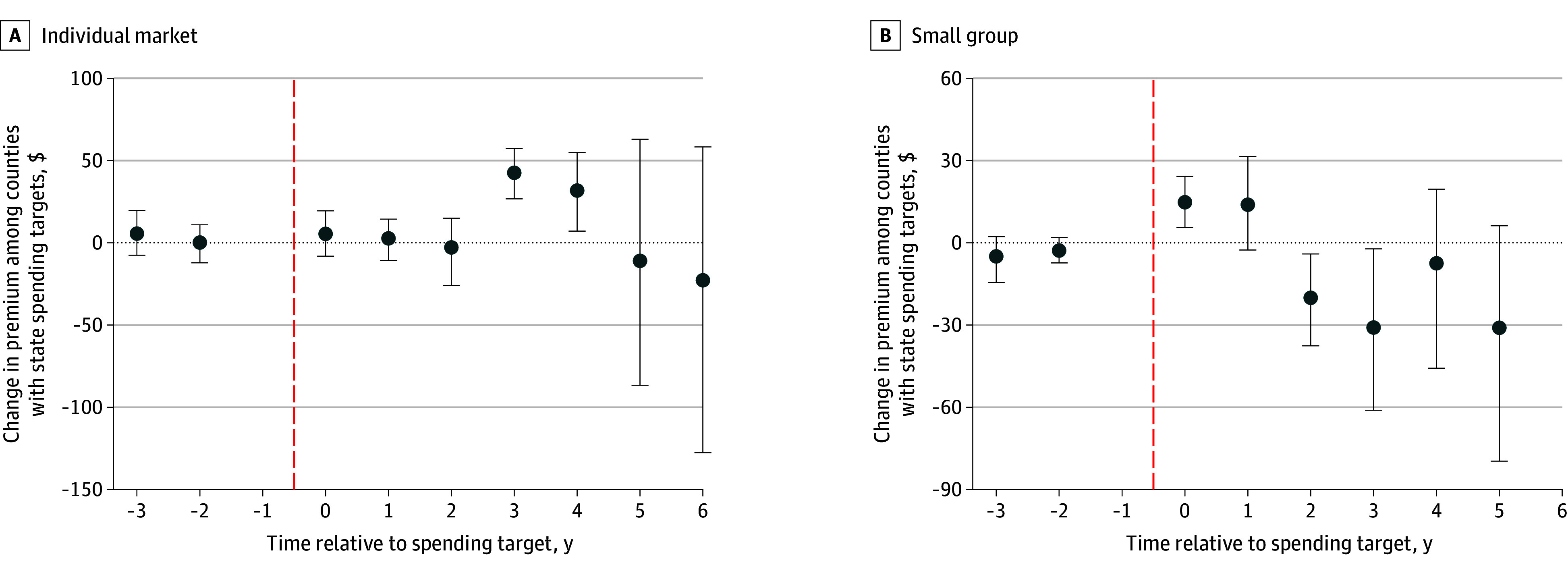
Changes in Individual Market and Small Group Bronze Premiums Before and After Benchmark Implementation Relative to Comparators Estimates for later years are based only on the subset of treated states with sufficient follow-up data. Outcome of interest is the monthly single premium for a patient aged 27 years. Error bars indicate 95% CIs; vertical dashed lines indicate implementation.

We analyzed individual market premiums in 184 treatment and 2924 comparison counties and small group premiums in 126 treatment and 2982 comparison counties (Figure 3). For the individual market, we found no associations except for in years 3 and 4 after implementation, during which benchmarks were associated with monthly premiums increases of as much as $43 (95% CI, $27-$57) (overall change, ($8 [95% CI, −$8 to $26]). In the small group market, benchmarks were associated with a $15 (95% CI, $6-$24) premium increase immediately after they took effect, but this association subsequently declined by as much as $31 (95% CI, −$61 to −$2) relative to comparators in year 3 after implementation (overall change, $11 [95% CI, −$3 to $25]).

###  State-Specific Analyses

In addition to the combined regressions shown above, we conducted separate regressions for each treatment state. Table 2 shows the mean findings across year, with additional detail presented in eFigures 3 to 5 in Supplement 1. For most states, we found no association between benchmarks and the hospital outcomes considered. One exception was Rhode Island, in which benchmarks were associated with $55 (95% CI, −$88 to −$20) decrease in outpatient prices. In Delaware and New Jersey, we found negative associations of benchmarks with individual market premiums of −$49 (95% CI, −$124 to −$20) and −$59 (95% CI, −$108 to −$22), respectively, when the mean was calculated across all years. We found positive associations of benchmarks with individual market premiums in Oregon ($14 [95% CI, $3-$26]) and Washington ($34 [95% CI, $22-$48]). We also found a positive association with small group premiums in New Jersey ($117 [95% CI, $94-$138]) and a negative association with small group premiums in Oregon (−$12 [95% CI, −$26 to $0]).

**Table 2.** Results of State-Specific Analyses

State	Estimate (95% CI)^a^						
	Inpatient revenue per discharge	Outpatient revenue per discharge equivalent	Standardized inpatient price	Standardized outpatient price	Individual market bronze premium	Small group bronze premium
Vermont	$123 (−$2876 to $4213)	$699 (−$4552 to $7168)	−$7 (−$2217 to $2172)	−$15 (−$46 to $22)	NA	NA
Delaware	$939 (−$2062 to $5426)	−$680 (−$6069 to $3886)	$4516 (−$2702 to $10 068)	$21 (−$17 to $58)	−$49 (−$124 to −$20)^b^	−$5 (−$47 to $18)
Rhode Island	−$2330 (−$5235 to $456)	−$1296 (−$6109 to $1925)	$596 (−$2211 to $3660)	−$55 (−$88 to −$20)^b^	$8 (−$33 to $48)	−$5 (−$27 to $16)
Connecticut	−$103 (−$9589 to $9459)	$452 (−$4551 to $6030)	−$1324 (−$6816 to $3838)	$14 (−$17 to $43)	$32 (−$16 to $63)	−$72 (−$112 to $1)
Oregon	−$4861 (−$18 477 to $1261)	−$73 (−$4624 to $3448)	$839 (−$1400 to $2964)	$23 (−$26 to $62)	$14 ($3 to $26)^b^	−$12 (−$26 to $0)^b^
Washington	−$62 (−$7473 to $7259)	$953 (−$2358 to $4673)	−$2057 (−$9949 to $7156)	$17 (−$20 to $64)	$34 ($22 to $48)^b^	−$3 (−$20 to $14)
New Jersey	$688 (−$1883 to $3842)	$635 (−$1027 to $2315)	NA	NA	−$59 (−$108 to −$22)^b^	$117 ($94 to $138)^b^
California	NA	NA	NA	NA	$12 (−$28 to 56)	NA
Overall	−$839 (−$4276 to $1874)	$439 (−$1207 to $2126)	−$3 (−$2290 to $2417)	$11 (−$12 to $34)	$8 (−$8 to $26)	$11 (−$3 to $25)

Abbreviation: NA, not applicable.

^a^ Estimates reflect the mean association between the outcome and benchmark implementation across all postintervention years observed for the state and outcome.

^b^ Indicates *P* < .05.

### Other Sensitivity Analyses

We conducted several additional sensitivity analyses, including running unweighted analyses, using a premium index (rather than premium levels) for individual and small group premiums, dropping covariates from our regressions, winsorizing the hospital revenue measures to reduce the influence of outliers, limiting the RHD analysis to short-term, general stay hospitals, and reporting premiums analyses that include Vermont. These results are presented in eTables 7 to 12 and eFigures 6 to 10 in Supplement 1. None of these analyses changed the conclusion that spending growth benchmarks had no association with hospital prices and net patient revenue, and mixed associations with premiums (eMethods 4 in Supplement 1). Event studies for the main results can be found in eTables 13 to 18 in Supplement 2.

## Discussion

In this case-control study, state spending growth benchmarks had little to no association with changes in hospital revenue or prices. Associations with premiums were inconsistent, with benchmarks being associated with premium increases in some years. We are reluctant to interpret these premium changes as causal for several reasons. First, associations with increases in individual market premiums were often coupled with associations with decreases in small group premiums, suggesting that adverse selection from the small group to the individual market may be contributing to results. Second, in response to the COVID-19 pandemic, federal policymakers implemented enhanced marketplace subsidies, which contributed to a doubling of enrollment between 2020 and 2025.^20^ Although the subsidy enhancements were the same in all states, our regressions required a fairly strong assumption that the risk composition of new enrollees was similar in treated and untreated communities.

The lack of associations with hospital prices and revenue, coupled with inconclusive premium results, suggest that state health care cost benchmarks have done little to curtail spending growth. One possible reason for the limited evidence is that benchmarks were not strongly enforced. Of the 8 treatment states that we analyzed, only 3—Vermont, Oregon, and California—had enforcement mechanisms. However, Vermont’s mechanism, which enabled CMS to set the benchmark if the state’s approach failed, may not have created strong incentives to comply.^21^ Both California and Oregon have stronger enforcement mechanisms, enabling them to name health care institutions and payers with growth that exceeds the benchmark, institute performance improvement plans, and levy fines. However, because California’s program is very new, these data are included in our analysis for only 1 outcome year (2025 individual market premiums).

Our results for Oregon mirrored our results for other states despite Oregon’s stronger enforcement approach. For the performance period from 2021 to 2022, Oregon identified 28 payers and providers with spending growth above the benchmark.^22^ However, the state determined that 25 of these entities had acceptable reasons for high spending growth. Because fines will only be levied on entities that had unacceptable performance in 3 of the past 5 years, the high rate of acceptable determinations suggests that few fines will be levied when they take effect in 2026. Infrequent enforcement has been the pattern in Massachusetts, which has exercised its authority to take corrective action only once.^1,23^ History suggests that limited enforcement could be a serious threat to the ability of spending growth benchmarks to achieve their goals; for example, a 1978 policy enabling voluntary limits on hospital spending growth collapsed by 1981.^24^ The Congressional Budget Office estimated that national-level policies similar to state spending growth benchmarks could be part of effective cost containment if coupled with strong enforcement,^25^ but it is not clear that this condition is currently being met.

It is possible that we might see negative associations over a longer period of time. However, these analyses have 5 years of follow-up data for hospital prices, 6 years of follow-up data for hospital revenue, and 7 years of follow-up data for premiums. None of these analyses suggest a trend toward reduced spending. These results imply that policymakers should not expect short-term savings with benchmarks.

### Limitations

This study has some limitations. Because of variation in implementation dates and lags in data availability, some states have few years of postimplementation data. Observations beyond 4 years come primarily from Vermont, Delaware, and Rhode Island. Additionally, the onset of the coronavirus pandemic occurred shortly after benchmark implementation in several states (Vermont, Delaware, and Rhode Island) and in the immediate preintervention period for others (Connecticut, Oregon, and Washington). We controlled for COVID-19 case rates and death rates, and because the coronavirus affected all states, it is not clear that the pandemic would bias our regressions. However, if certain outcomes such as postpandemic pent-up demand for health services were different in treatment and comparison states, our results could be affected.

Many states implemented other policies during the past 10 years to address rising health care spending. For example, starting in 2010, Rhode Island implemented a suite of affordability standards that included a cap on hospital price growth for fully insured commercial plans,^26,27^ and starting in 2019 Oregon implemented a hospital payment cap in its state employee benefits plan.^28^ While these policy changes predated implementation of spending growth benchmarks, lagged effects or regulatory adjustments could have occurred in the postbenchmark period. More generally, it is hard to compare states given differences in regulatory, policy, and economic conditions that vary over time. We used EB, DD, and control variables to address this issue; regardless, some factors may remain unmeasured.

Our 3 outcomes each have strengths and weaknesses. HCRIS data represent all hospitals that receive Medicare reimbursement but do not include true measures of prices. HPTD data contain prices but come from a limited sample of commercial claims. Future analyses may consider using other claims data sources that represent a larger share of the commercial market. Both HPTD and HCRIS are limited to hospital data only. Individual and small group premiums capture all health care spending but only for a subset of individuals enrolled in these markets. These premium measures may also be affected by state policy decisions that affect spending (such as Vermont’s Act 111,^29^ which limited health plans’ use of prior authorization) and local economic conditions may affect who enrolls and insurer participation decisions.

## Conclusions

The findings of this case-control study suggest that health care spending benchmarks have not achieved their goal of reducing spending growth. To achieve an impact, benchmarks may need to be paired with stricter enforcement mechanisms or other policies, such as price caps or reference pricing.

## Notes

Supplement 1. **eTable 1.** State Spending Growth Targets and Actual Growth **eTable 2.** List of Variables Included in Analysis **eTable 3.** Rate of Missing Data **eFigure 1.** Exclusions Made When Constructing Hospital-Level Analytic File **eFigure 2.** Exclusions Made When Constructing County-Level Analytic File **eTable 4.** Treatment and Control Status of US States in the Analysis **eMethods 1.** Additional Data Sources **eMethods 2.** Additional Details on Multiple Imputation **eMethods 3.** Inference **eTable 5.** Effective Sample Sizes from Entropy Balancing **eTable 6.** Distribution of Entropy Balancing Weight Size **eFigure 3.** State-Specific Event-Study Plots for Hospital Revenue Outcomes **eFigure 4.** State-Specific Event-Study Plots for Hospital Price Outcomes **eFigure 5.** State-Specific Event-Study Plots for Premiums Outcomes **eMethods 4.** Sensitivity Analyses **eTable 7.** Sensitivity Analyses of State-Specific Overall Effect Estimates for Inpatient Hospital Revenue per Discharge **eTable 8.** Sensitivity Analyses of State-Specific Overall Effect Estimates for Outpatient Hospital Revenue per Discharge Equivalent **eFigure 6.** Sensitivity Analyses of Event-Study Plots for Hospital Revenue Outcomes **eTable 9.** Sensitivity Analyses of State-Specific Overall Effect Estimates for Inpatient Hospital Price **eTable 10.** Sensitivity Analyses of State-Specific Overall Effect Estimates for Outpatient Hospital Price **eFigure 7.** Sensitivity Analyses of Event-Study Plots for Hospital Price Outcomes **eTable 11.** Sensitivity Analyses of State-Specific Overall Effect Estimates for Individual Market Premium **eTable 12.** Sensitivity Analyses of State-Specific Overall Effect Estimates for Small Group Premium **eFigure 8.** Sensitivity Analyses of Event-Study Plots for Premium Outcomes **eFigure 9.** Event-Study Plots for Indexed Premium Outcomes **eFigure 10.** Event-Study Plots for Premium Outcomes, Including Vermont

Supplement 2. **eTable 13.** Event-Study Estimates for Inpatient Hospital Revenue per Discharge **eTable 14.** Event-Study Estimates for Outpatient Hospital Revenue per Discharge Equivalent **eTable 15.** Event-Study Estimates for Inpatient Hospital Price **eTable 16.** Event-Study Estimates for Outpatient Hospital Price **eTable 17.** Event-Study Estimates for Individual Market Premium **eTable 18.** Event-Study Estimates for Small Group Premium
eReferences

Supplement 3.
Data Sharing Statement
