# Supplementary material for: State Spending Growth Benchmarks and Hospital Revenue, Hospital Prices, and Premiums
Source: JAMA Netw Open. 2026 Feb 20;9(2):e2558283. doi: 10.1001/jamanetworkopen.2025.58283 (PMC12924095; doi:10.1001/jamanetworkopen.2025.58283)
Supplement: Supplement 1. — eTable 1. State Spending Growth Targets and Actual Growth eTable 2. List of Variables Included in Analysis eTable 3. Rate of Missing Data eFigure 1. Exclusions Made When Constructing Hospital-Level Analytic File eFigure 2. Exclusions Made When Constructing County-Level Analytic File eTable 4. Treatment and Control Status of US States in the Analysis eMethods 1. Additional Data Sources eMethods 2. Additional Details on Multiple Imputation eMethods 3. Inference eTable 5. Effective Sample Sizes from Entropy Balancing eTable 6. Distribution of Entropy Balancing Weight Size eFigure 3. State-Specific Event-Study Plots for Hospital Revenue Outcomes eFigure 4. State-Specific Event-Study Plots for Hospital Price Outcomes eFigure 5. State-Specific Event-Study Plots for Premiums Outcomes eMethods 4. Sensitivity Analyses eTable 7. Sensitivity Analyses of State-Specific Overall Effect Estimates for Inpatient Hospital Revenue per Discharge eTable 8. Sensitivity Analyses of State-Specific Overall Effect Estimates for Outpatient Hospital Revenue per Discharge Equivalent eFigure 6. Sensitivity Analyses of Event-Study Plots for Hospital Revenue Outcomes eTable 9. Sensitivity Analyses of State-Specific Overall Effect Estimates for Inpatient Hospital Price eTable 10. Sensitivity Analyses of State-Specific Overall Effect Estimates for Outpatient Hospital Price eFigure 7. Sensitivity Analyses of Event-Study Plots for Hospital Price Outcomes eTable 11. Sensitivity Analyses of State-Specific Overall Effect Estimates for Individual Market Premium eTable 12. Sensitivity Analyses of State-Specific Overall Effect Estimates for Small Group Premium eFigure 8. Sensitivity Analyses of Event-Study Plots for Premium Outcomes eFigure 9. Event-Study Plots for Indexed Premium Outcomes eFigure 10. Event-Study Plots for Premium Outcomes, Including Vermont [file jamanetwopen-e2558283-s001.pdf]

Supplementary Online Content

Eibner C, Chase EC, Kerber R, Liu JL. State spending growth benchmarks and hospital revenue, hospital prices, and premiums. *JAMA Netw Open.* 2026;9(2):e2558283. doi:10.1001/jamanetworkopen.2025.58283

eTable 1. State Spending Growth Targets and Actual Growth

eTable 2. List of Variables Included in Analysis

eTable 3. Rate of Missing Data

eFigure 1. Exclusions Made When Constructing Hospital-Level Analytic File

eFigure 2. Exclusions Made When Constructing County-Level Analytic File

eTable 4. Treatment and Control Status of US States in the Analysis

eMethods 1. Additional Data Sources

eMethods 2. Additional Details on Multiple Imputation

eMethods 3. Inference

eTable 5. Effective Sample Sizes from Entropy Balancing

eTable 6. Distribution of Entropy Balancing Weight Size

eFigure 3. State-Specific Event-Study Plots for Hospital Revenue Outcomes

eFigure 4. State-Specific Event-Study Plots for Hospital Price Outcomes

eFigure 5. State-Specific Event-Study Plots for Premiums Outcomes

eMethods 4. Sensitivity Analyses

eTable 7. Sensitivity Analyses of State-Specific Overall Effect Estimates for Inpatient Hospital Revenue per Discharge

eTable 8. Sensitivity Analyses of State-Specific Overall Effect Estimates for Outpatient Hospital Revenue per Discharge Equivalent

eFigure 6. Sensitivity Analyses of Event-Study Plots for Hospital Revenue Outcomes

eTable 9. Sensitivity Analyses of State-Specific Overall Effect Estimates for Inpatient Hospital Price

© 2026 Eibner C et al. *JAMA Network Open*.

eTable 10. Sensitivity Analyses of State-Specific Overall Effect Estimates for Outpatient Hospital Price

eFigure 7. Sensitivity Analyses of Event-Study Plots for Hospital Price Outcomes

eTable 11. Sensitivity Analyses of State-Specific Overall Effect Estimates for Individual Market Premium

eTable 12. Sensitivity Analyses of State-Specific Overall Effect Estimates for Small Group Premium

eFigure 8. Sensitivity Analyses of Event-Study Plots for Premium Outcomes

eFigure 9. Event-Study Plots for Indexed Premium Outcomes

eFigure 10. Event-Study Plots for Premium Outcomes, Including Vermont

eTable 13. Event-Study Estimates for Inpatient Hospital Revenue per Discharge

eTable 14. Event-Study Estimates for Outpatient Hospital Revenue per Discharge Equivalent

eTable 15. Event-Study Estimates for Inpatient Hospital Price

eTable 16. Event-Study Estimates for Outpatient Hospital Price

eTable 17. Event-Study Estimates for Individual Market Premium

eTable 18. Event-Study Estimates for Small Group Premium

eReferences

This supplementary material has been provided by the authors to give readers additional information about their work.

© 2026 Eibner C et al. *JAMA Network Open*.

eTable 1. State Spending Growth Targets and Actual Growth

State	Year(s)	Target	Actual
Massachusetts[1]	2013	3.6%	**2.4%**
	2014	3.6%	4.2%
	2015	3.6%	4.8%
	2016	3.6%	**3.0%**
	2017	3.6%	**2.8%**
	2018	3.1%	3.6%
	2019	3.1%	4.1%
	2020	3.1%	**-2.3%**
	2021	3.1%	9.0%
	2022	3.1%	5.8%
	2023	3.6%	8.6%
	2024-2026	3.6%	N/A
Vermont[a,2]	2018	3.5-4.3%	**3.8%**
	2019	3.5-4.3%	5.3%
	2020	3.5-4.3%	**-7.4%**
	2021	3.5-4.3%	16.5%
	2022	3.5-4.3%	**1.8%**
	2023	3.5-4.3%	10.3%
	2024	3.5-4.3%	N/A
Delaware[3]	2019	3.8%	5.8%
	2020	3.5%	**-1.1%**
	2021	3.25%	11.2%
	2022	3.0%	6.3%
	2023	3.1%	9.1%
	2024	3.0%	N/A
Rhode Island[4]	2019	3.2%	4.1%
	2020	3.2%	**-2.9%**
	2021	3.2%	**3.2%**
	2022	3.2%	**1.6%**
	2023	6.0%	7.8%
	2024	5.1%	N/A
	2025	3.6%	N/A
	2026-2027	3.3%	N/A
Connecticut[5]	2021	3.4%	6.0%
	2022	3.2%	3.4%
	2023	2.9%	7.9%
	2024	4.0%	N/A
	2025	2.9%	N/A
Oregon[b,6]	2021	3.4%	3.5%
	2022	3.4%	4.8%
	2023	3.4%	5.2%
	2024-2025	3.4%	N/A
	2026-2030	3.0%	N/A
Washington[7]	2022	3.2%	5.3%
	2023	3.2%	N/A
	2024	3.0%	N/A
	2025	3.0%	N/A
	2026	2.8%	N/A
New Jersey[8]	2023	3.5%	N/A
	2024	3.2%	N/A
	2025	3.9%	N/A
	2026	2.8%	N/A

© 2026 Eibner C et al. *JAMA Network Open*.

State	Year(s)	Target	Actual
	2027	2.8%	N/A
California[9]	2025	3.5%	N/A
	2026	3.5%	N/A
	2027	3.2%	N/A
	2028	3.2%	N/A
	2029+	3.0%	N/A

[a]Vermont also has a goal of 3.5% on average over 2018-2022.
[b]Oregon's targets for 2026-2030 may be adjusted in 2024 if needed.
NOTE: Nine states have enacted state spending growth benchmarks as of 2025. Years are calendar years.

© 2026 Eibner C et al. *JAMA Network Open*.

eTable 2. List of Variables Included in Analysis

Variable	Values	Hospital-Level	County-Level
Treatment Variables			
State	U.S. states	X	X
State healthcare spending target status	1 = In state with a healthcare spending target<br>0 = In state without a healthcare spending target	X	X
Date of spending target implementation	2018-2025, for states with spending targets	X	X
Outcome Variables			
Inpatient net patient revenue per discharge	Continuous	X	NA
Outpatient net patient revenue per discharge equivalent	Continuous	X	NA
Mean standardized inpatient hospital price	Continuous	NA	X
Mean standardized outpatient hospital price	Continuous	NA	X
Mean individual market 27-year-old bronze premium	Continuous	NA	X
Mean small group 27-year-old bronze premium	Continuous	NA	X
Entropy Balancing Variables			
Rural/urban status	0 = metropolitan area (RUCC 1-3)<br>1 = non-metropolitan area (RUCC 4+)	For county in which hospital is located	X
Median age	Continuous	For county in which hospital is located	X
Percent of population 18-64 with health insurance	Continuous	For county in which hospital is located	X
Hospital market concentration (HHI)	Continuous	For county in which hospital is located	X
Hospital government ownership status	0 = not government owned<br>1 = government owned	X	NA
Critical access hospital	0 = Not a critical access hospital<br>1 = Critical access hospital	X	NA
Short-term general hospital (STGH)	0 = Not a STGH<br>1 = STGH	X	NA
Number of hospital beds	Continuous	X	X
Entropy Balancing and Time-Varying Control Variables			
Total population	Continuous	For county in which hospital is located	X

© 2026 Eibner C et al. *JAMA Network Open*.

Variable	Values	Hospital-Level	County-Level
Medicaid expansion status	1 = In state that had expanded Medicaid<br>0 = In state that had not expanded Medicaid	X	X
Time-Varying Control Variables			
Poverty rate	Continuous	For county in which hospital is located	X
Unemployment rate	Continuous	For county in which hospital is located	X
Percent of population age 65+	Continuous	For county in which hospital is located	X
COVID incidence rate	Continuous	For county in which hospital is located	X
COVID mortality rate	Continuous	For county in which hospital is located	X
Percent of inpatient insurance claims coming from that state's APCD	Continuous	NA	X
Percent of outpatient insurance claims coming from that state's APCD	Continuous	NA	X
Other Variables			
Number of discharges	Continuous	X	NA
Number of outpatient discharge equivalents	Continuous	X	NA
Number of individual market insurance carriers	Continuous	NA	X
Number of small group insurance carriers	Continuous	NA	X
Median household income	Continuous	For county in which hospital is located	X
Percent of population male	Continuous	For county in which hospital is located	X

© 2026 Eibner C et al. *JAMA Network Open*.

eTable 3. Rate of Missing Data

Variable	Percent Missing (Hospital Level)	Percent Missing (County Level)
Treatment Variables		
State	0%	0%
State healthcare spending target status	0%	0%
Date of spending target implementation	0%	0%
Outcome Variables		
Inpatient net patient revenue per discharge	2010-2023: 1.2%	NA
Outpatient net patient revenue per discharge equivalent	2010-2023: 8.3%	NA
Mean standardized inpatient hospital price	NA	Among counties with at least 11 inpatient claims per year, 2016-2022: 0%
Mean standardized outpatient hospital price	NA	Among counties with at least 11 outpatient claims per year, 2016-2022: 0%
Mean individual market 27-year-old bronze premium	NA	2015-2025: 0.8%
Mean small group 27-year-old bronze premium	NA	2015-2024: 1.1%
Entropy Balancing Variables		
Rural/urban status	<0.1%	<0.1%
Median age	2010-2023: 0.2%	2010-2023: 0.1%
Percent of population 18-64 with health insurance	2013-2019: 2.0%	2013-2019: 26.1%
Hospital market concentration (HHI)	2010-2023: 1.0%	2010-2023: 25.9%
Hospital government ownership status	2010-2023: 0%	NA
Critical access hospital	2010-2023: 0%	NA
Short-term general hospital (STGH)	2010-2023: 0%	NA
Number of hospital beds	2010-2023: 0.1%	2010-2023: 25.9%
Entropy Balancing and Time-Varying Control Variables		
Total population	2010-2023: 0.2%	2010-2023: 0.1%
Medicaid expansion status	0%	0%
Time-Varying Control Variables		
Poverty rate	2010-2023: 0.1%	2010-2023: 0.2%
Unemployment rate	2010-2023: <0.1%	2010-2023: 0.1%
Percent of population age 65+	2010-2023: 0.2%	2010-2023: 0.1%
COVID incidence rate	2020-2022: 0.5%	2020-2022: 0.2%
COVID mortality rate	2020-2022: 0.5%	2020-2022: 0.2%
Percent of inpatient insurance claims coming from that state’s APCD	NA	Among counties with at least 11 inpatient claims per year, 2016-2022: 0%
Percent of outpatient insurance claims coming from that state’s APCD	NA	Among counties with at least 11 outpatient claims per year, 2016-2022: 0%
Other Variables		
Number of discharges	2010-2023: 0.2%	NA
Number of outpatient discharge equivalents	2010-2023: 0.1%	NA
Number of individual market insurance carriers	NA	2015-2025: 0.8%
Number of small group insurance carriers	NA	2015-2024: 1.1%
Median household income	2010-2023: 0.1%	2010-2023: 0.2%
Percent of population male	2010-2023: 0.2%	2010-2023: 0.1%

© 2026 Eibner C et al. *JAMA Network Open*.

eFigure 1. Exclusions Made When Constructing Hospital-Level Analytic File

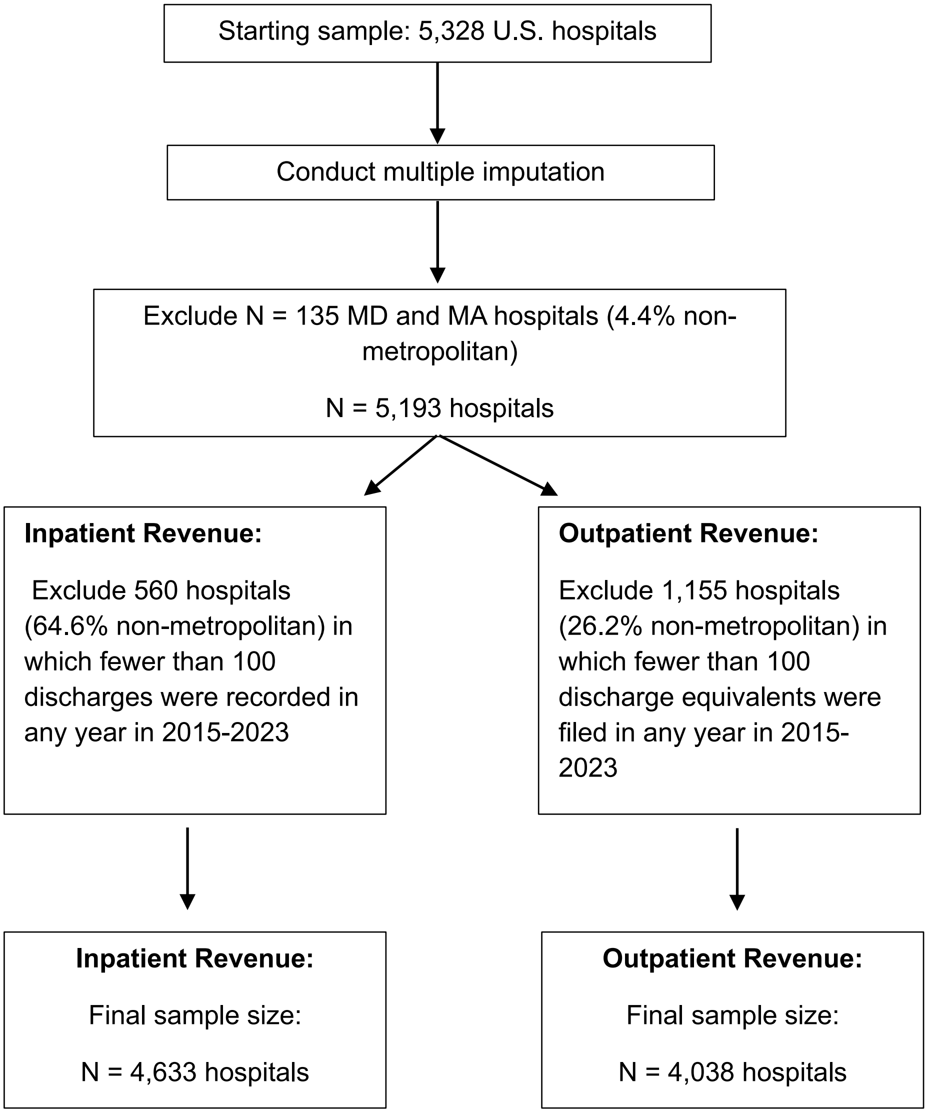

© 2026 Eibner C et al. *JAMA Network Open*.

eFigure 2. Exclusions Made When Constructing County-Level Analytic File

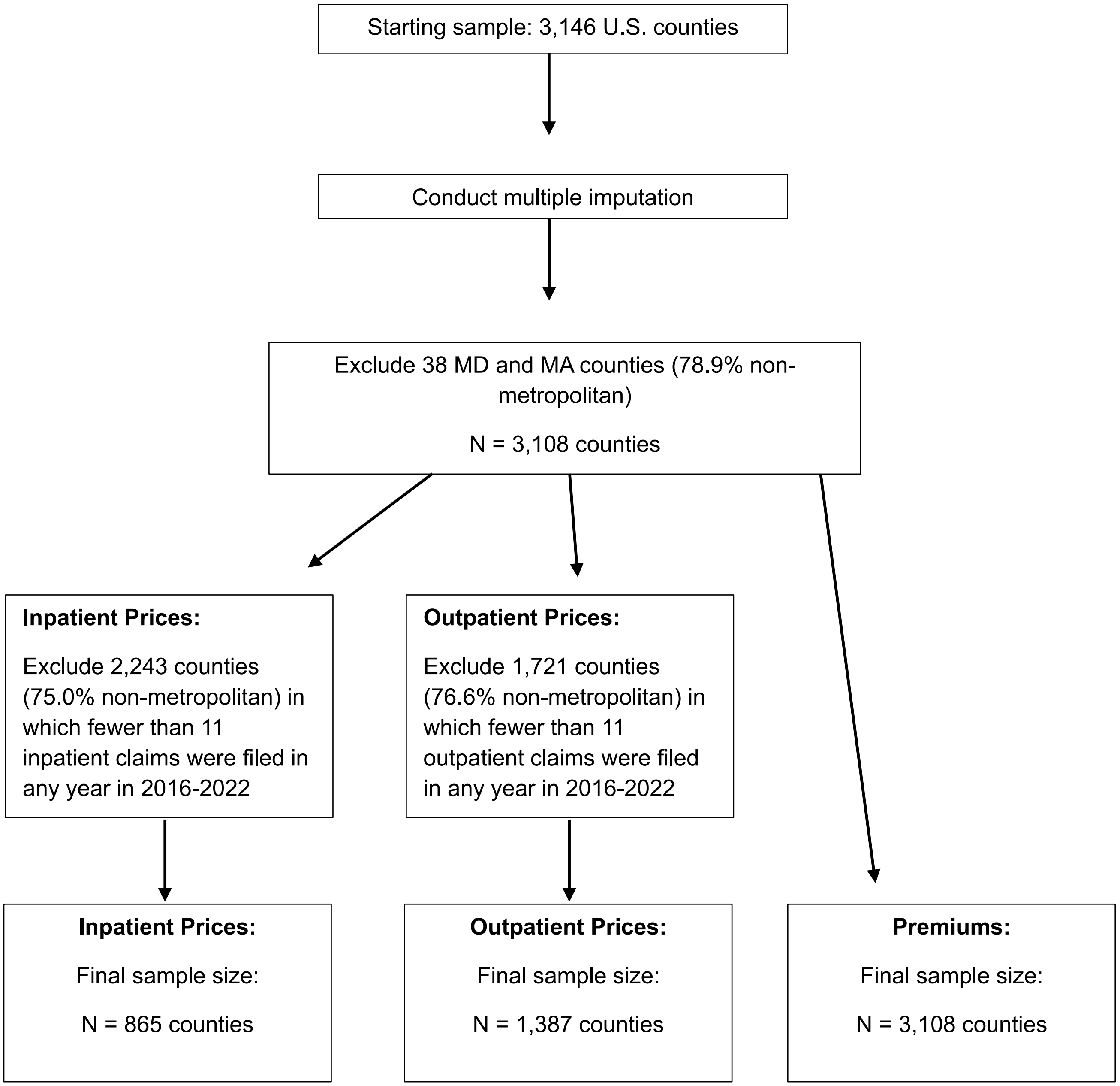

© 2026 Eibner C et al. *JAMA Network Open*.

eTable 4. Treatment and Control Status of US States in the Analysis

Group	States	Number of Hospitals	Number of Counties
Treated – Hospital Revenue	VT, DE, RI, CT, OR, WA, NJ	298	-
Control – Hospital Revenue	AL, AK, AZ, AR, CA, CO, FL, GA, HI, ID, IL, IN, IA, KS, KY, LA, ME, MI, MN, MS, MO, MT, NE, NV, NH, NM, NY NC, ND, OH, OK, PA, SC, SD, TN, TX, UT, VA, WV, WI, WY	4,515	-
Treated – Hospital Prices	VT, DE, RI, CT, OR, WA	-	89
Control – Hospital Prices	AL, AK, AZ, AR, CA, CO, FL, GA, HI, ID, IL, IN, IA, KS, KY, LA, ME, MI, MN, MS, MO, MT, NE, NJ, NV, NH, NM, NY NC, ND, OH, OK, PA, SC, SD, TN, TX, UT, VA, WV, WI, WY	-	1,316
Treated – IM Premiums	VT, DE, RI, CT, OR, WA, NJ, CA	-	184
Control – IM Premiums	AL, AK, AZ, AR, CO, FL, GA, HI, ID, IL, IN, IA, KS, KY, LA, ME, MI, MN, MS, MO, MT, NE, NV, NH, NM, NY NC, ND, OH, OK, PA, SC, SD, TN, TX, UT, VA, WV, WI, WY	-	2,924
Treated – SG Premiums	VT, DE, RI, CT, OR, WA, NJ	-	126
Control – SG Premiums	AL, AK, AZ, AR, CA, CO, FL, GA, HI, ID, IL, IN, IA, KS, KY, LA, ME, MI, MN, MS, MO, MT, NE, NV, NH, NM, NY NC, ND, OH, OK, PA, SC, SD, TN, TX, UT, VA, WV, WI, WY	-	2,982
Excluded from All Analyses	MA, MD	135	38

© 2026 Eibner C et al. *JAMA Network Open*.

eMethods 1. Additional Data Sources

In addition to the RAND Hospital Data (RHD), the RAND Hospital Price Transparency Data (HPTD), and the premiums data that we used in our analyses of health care spending outcomes, we also assembled data on other variables that we used as covariates in the entropy balancing (EB) and difference in differences (DD) models. These data comprised:

- U.S. Census Bureau estimates of the total and age/sex-stratified population at the county level for 2010-2023[10]
- The U.S. Census Bureau's Small Area Income and Poverty Estimates data[11]: annual county-level estimates of the poverty rate and medium household income for 2010-2023.
- The U.S. Bureau of Labor Statistics' annual county-level estimates of the unemployment rate for 2010-2023.[12]
- The Centers for Disease Control and Prevention county-level estimates of COVID-19 incidence and mortality for 2020-2022.[13]
- The U.S. Department of Agriculture Economic Research Service's county-level rural/urban classifications for 2010 and 2020.[14]
- The Health Resources and Services Administration's Area Health Resource File county-level estimates of the percent of the population ages 18-64 with health insurance for 2013-2019.[15]

Using these data sources and the data described in the main manuscript, we assembled two analytic files:

- A county-level file from 2010 to 2025 that contained the variables listed above, along with the county-level HPTD and premiums outcomes, and variables derived from the RHD, such as the number of hospital beds in the county or the county hospital market concentration.
- A hospital-level file from 2010 to 2023 that contained the RHD outcomes, variables derived from the RHD such as number of hospital beds, and the variables listed above (we used the county-level variable for the county in which each hospital was located).

Note that we did not directly use any data prior to 2015 in any of the analyses; we included this earlier data to support the multiple imputation, which is described further below. A complete list of variables is given in eTable 3.

eMethods 2. Additional Details on Multiple Imputation

There was some missing data in our analytic files. While most of this missing data was sporadic and minor, there were several challenging concerns:

- *COVID-19 data for 2023-2025*: While COVID-19 data was available for 2020-2022, and we were comfortable assuming that COVID-19 incidence and mortality was zero prior to 2020, we did not have access to county-level COVID-19 incidence and mortality data for 2023-2025. We needed this data for 2023 for the RHD outcomes, and for 2024-2025 for the premium outcomes. Using 2020-2022 data to impute COVID incidence and mortality in 2023-2025 is questionable, because the multiple imputation may not account for the decrease in COVID mortality in 2023-2025 due to vaccines and acquired immunity. As a result, we conduct sensitivity analyses in which all covariates (including the COVID information) are omitted from the modeling.
- *Percent of the 18-64 population with health insurance for 2020-2025*: This variable was only available through 2019, but we needed it through 2021 for the HPTD outcomes, through 2022 for the RHD outcomes, and through 2024 for the premium outcomes.
- *Census and federal data sources for 2024-2025*: None of the data from federal sources (Census data, BLS unemployment data, USDA rurality data) was available beyond 2023, but we needed these data in 2024-2025 for the premium outcomes.
- *RHD outcomes data*: The CMS cost reports underlying the RHD have some issues with data quality and reporting, which have been documented elsewhere.[16,17] We decided to set some of the RHD outcomes to missing (and then impute replacement values) when the following occurred:

© 2026 Eibner C et al. *JAMA Network Open*.

- We set the number of inpatient discharges to missing if the reported number of inpatient discharges was less than one.
- We set the number of discharge equivalents to missing if the reported number of discharge equivalents was less than zero.
- We set inpatient revenue per discharge and outpatient revenue per discharge to missing if it was negative or greater than the 99th percentile for this variable.

eTable 4 provides information on the rate of missingness for all of our predictors and outcomes. We used multiple imputation to address the missing data.[18]

For the hospital-level analytic file, we included the following variables in the multiple imputation for years 2010-2023: hospital and state fixed effects, year, inpatient revenue per discharge, outpatient net patient revenue per discharge equivalent, number of discharges, number of discharge equivalents, government ownership status, critical access hospital status, short-term general hospital status, poverty rate, median household income, unemployment rate, county population, number of hospital beds, median age, percent of the population male, percent of the population age 65+, percent of the population ages 18-64 with health insurance, rural/urban status, COVID-19 incidence rate, COVID-19 mortality rate, hospital market concentration, whether or not a healthcare spending target was in effect in that year, and whether or not Medicaid expansion was in effect in that year.

For the county-level analytic file, we included the following variables in the multiple imputation for years 2010-2025: county and state fixed effects, year, mean individual market premium, mean small group premium, number of individual market carriers, number of small group carriers, mean 2015-indexed individual market premium, mean 2015-indexed small group premium, standardized inpatient price, standardized outpatient price, percent of inpatient claims coming from an all-payer claims database (APCD), percent of outpatient claims coming from an APCD, poverty rate, median household income, unemployment rate, county population, number of hospital beds, median age, percent of the population male, percent of the population age 65+, percent of the population ages 18-64 with health insurance, rural/urban status, COVID-19 incidence rate, COVID-19 mortality rate, hospital market concentration, whether or not the premiums use full community rating, whether or not a healthcare spending target was in effect in that year, and whether or not Medicaid expansion was in effect in that year.

We then carried out multiple imputation with predictive mean matching using the R package 'mice.' Note that our decision to include state, county/hospital, and year effects is consistent with guidance for imputing hierarchical data[19]; similarly, our decision to include the outcome variables in the multiple imputation is considered good practice.[20] We generated 50 imputations for each analytic file.

After conducting the imputation, we applied the following inclusion criteria on each imputation:

- For the RHD outcomes, we restricted the hospital-level analytic file to 2015-2023, removed data from Maryland and Massachusetts, and only included hospitals that had at least 100 inpatient discharges (for the inpatient outcome) or at least 100 outpatient discharge equivalents (for the outpatient outcome).
- For the HPTD outcomes, we restricted the county-level analytic file to 2015-2022, removed data from Maryland and Massachusetts, and only included counties that had at least 11 inpatient claims and at least 11 outpatient claims in the years of analysis.
- For the individual market premium outcome, we restricted the county-level analytic file to 2015-2025 and removed data from Maryland and Massachusetts.
- For the small group premium outcome, we restricted the county-level analytic file to 2015-2024 and removed data from Maryland and Massachusetts.

Applying these inclusion criteria *after* imputation (as we do here) is the recommended approach.[21] eFigures 1-2 show the exclusions made in constructing our analytic samples for the hospital- and county-level analyses, respectively. We discuss how inference was conducted across multiple imputations in the Inference section below.

eMethods 3. Inference

We used a fractionated bootstrap in combination with the multiple imputation to conduct inference for our estimated treatment effects.[18,22] Our inference routine was:

© 2026 Eibner C et al. *JAMA Network Open*.

1. Generate 50 multiple imputations (described above).
2. On each imputation:
    a. Apply exclusion criteria.
    b. Generate 250 bootstrap samples via fractionated weighting. On each bootstrap replicate:
        i. For each county/hospital, calculate a fractionated bootstrap weight.[22] Apply that fractionated bootstrap weight to all years for that county/hospital.
        ii. For each treated state:
            1. Carry out the EB to construct a comparison set of never-treated counties/hospitals.
            2. Calculate a final analytic weight as the product of the fractionated bootstrap weight, the EB weight, and the size weight (either county population or hospital beds).
            3. Run the DD models using the final analytic weight.
            4. Store these state-specific results.
        iii. Aggregate results across treated states by taking the weighted mean of the treatment effects proportional to the number of treated counties/hospitals in each treated state.[23,24] Store these state-aggregated results.

At the end of this routine, we have 50 imputations x 250 bootstrap samples = 12,500 estimates of the state-specific and state-aggregated treatment effects. We obtain point and confidence interval estimates for the treatment effects by taking the mean and 2.5th and 97.5th percentiles of the 12,500 samples.[25]

In the main manuscript, we presented the overall treatment effect estimate for each treated state and the event study plots aggregated across states. We present state-specific event study plots in eFigures 3-5. We also provide the specific numbers underlying these plots and the event study plots presented in the main manuscript as a supplemental Excel spreadsheet.

© 2026 Eibner C et al. *JAMA Network Open*.

eTable 5. Effective Sample Sizes from Entropy Balancing

State	Inpatient Net Patient Revenue Per Discharge	Outpatient Net Patient Revenue Per Discharge Equivalent	Inpatient Prices	Outpatient Prices	Individual Market Premium	Small Group Premium
**California**						
Treated N	NA	NA	NA	NA	58	NA
Control ESS	NA	NA	NA	NA	22	NA
					(16, 30)	
**Connecticut**						
Treated N	32	29	8	8	8	8
Control ESS	206	92	14	37	2	6
	(194, 213)	(86, 116)	(12, 16)	(25, 42)	(2, 2)	(5, 7)
**Delaware**						
Treated N	12	10	3	3	3	3
Control ESS	36	20	5	6	4	5
	(35, 36)	(20, 20)	(5, 5)	(6, 6)	(4, 4)	(5, 6)
**New Jersey**						
Treated N	84	63	NA	NA	21	21
Control ESS	211	261	NA	NA	8	6
	(187, 233)	(249, 272)			(8, 9)	(6, 7)
**Oregon**						
Treated N	58	56	29	27	36	36
Control ESS	674	614	51	59	149	65
	(632, 702)	(587, 638)	(48, 54)	(52, 64)	(147, 152)	(60, 72)
**Rhode Island**						
Treated N	12	11	4	4	5	5
Control ESS	43	42	3	7	5	6
	(43, 43)	(42, 47)	(3, 3)	(7, 7)	(4, 6)	(5, 7)
**Vermont**						
Treated N	14	15	12	12	NA	NA
Control ESS	63	54	17	33	NA	NA
	(62, 63)	(54, 55)	(17, 17)	(33, 33)		
**Washington**						
Treated N	85	83	15	31	39	39
Control ESS	627	633	59	166	213	20
	(576, 654)	(624, 641)	(54, 63)	(160, 175)	(205, 219)	(18, 23)

ESS = effective sample size
NOTE: The table shows the median (IQR) ESS of the control units across the 50 multiple imputations, because ESS varies across imputations.

© 2026 Eibner C et al. *JAMA Network Open*.

eTable 6. Distribution of Entropy Balancing Weight Size

State	Inpatient Net Patient Revenue Per Discharge	Outpatient Net Patient Revenue Per Discharge Equivalent	Inpatient Prices	Outpatient Prices	Individual Market Premium	Small Group Premium
**California**						
0	NA	NA	NA	NA	1.7%	NA
(0, 1)	NA	NA	NA	NA	94.8%	NA
[1, 10)	NA	NA	NA	NA	1.8%	NA
10+	NA	NA	NA	NA	1.6%	NA
**Connecticut**						
0	0.0%	0.5%	1.5%	0.1%	13.9%	21.3%
(0, 1)	89.0%	90.5%	92.1%	90.3%	85.4%	78.2%
[1, 10)	8.2%	6.4%	4.4%	7.5%	0.2%	0.1%
10+	2.8%	2.6%	2.1%	2.2%	0.5%	0.4%
**Delaware**						
0	2.5%	2.4%	21.8%	10.4%	13.7%	34.2%
(0, 1)	94.4%	93.7%	72.3%	86.6%	85.9%	65.4%
[1, 10)	1.8%	2.8%	1.7%	1.2%	0.1%	0.1%
10+	1.3%	1.1%	4.2%	1.8%	0.3%	0.4%
**New Jersey**						
0	2.0%	1.9%	NA	NA	4.8%	59.5%
(0, 1)	84.6%	83.2%	NA	NA	94.2%	40.2%
[1, 10)	10.7%	12.0%	NA	NA	0.4%	0.0%
10+	2.7%	2.9%	NA	NA	0.6%	0.3%
**Oregon**						
0	0.1%	0.0%	0.9%	0.0%	0.1%	1.4%
(0, 1)	72.8%	73.4%	85.1%	89.3%	88.0%	93.4%
[1, 10)	26.1%	25.5%	11.3%	8.1%	9.3%	2.7%
10+	1.0%	1.1%	2.7%	2.6%	2.6%	2.6%
**Rhode Island**						
0	2.1%	2.2%	27.7%	3.9%	50.7%	34.6%
(0, 1)	91.2%	91.3%	66.4%	92.7%	48.9%	65.0%
[1, 10)	4.5%	4.5%	4.2%	0.8%	0.1%	0.1%
10+	2.2%	1.9%	1.7%	2.6%	0.3%	0.4%
**Vermont**						
0	5.6%	9.0%	0.0%	0.4%	NA	NA
(0, 1)	86.6%	83.1%	82.4%	86.6%	NA	NA
[1, 10)	5.7%	5.8%	16.0%	10.2%	NA	NA
10+	2.1%	2.1%	1.7%	2.8%	NA	NA
**Washington**						
0	0.0%	0.0%	0.0%	0.0%	0.0%	10.0%
(0, 1)	73.1%	71.3%	85.0%	78.9%	83.4%	87.5%
[1, 10)	25.9%	27.8%	12.5%	19.4%	14.4%	1.2%
10+	1.0%	0.9%	2.5%	1.7%	2.2%	1.2%

© 2026 Eibner C et al. *JAMA Network Open*.

eFigure 3. State-Specific Event-Study Plots for Hospital Revenue Outcomes

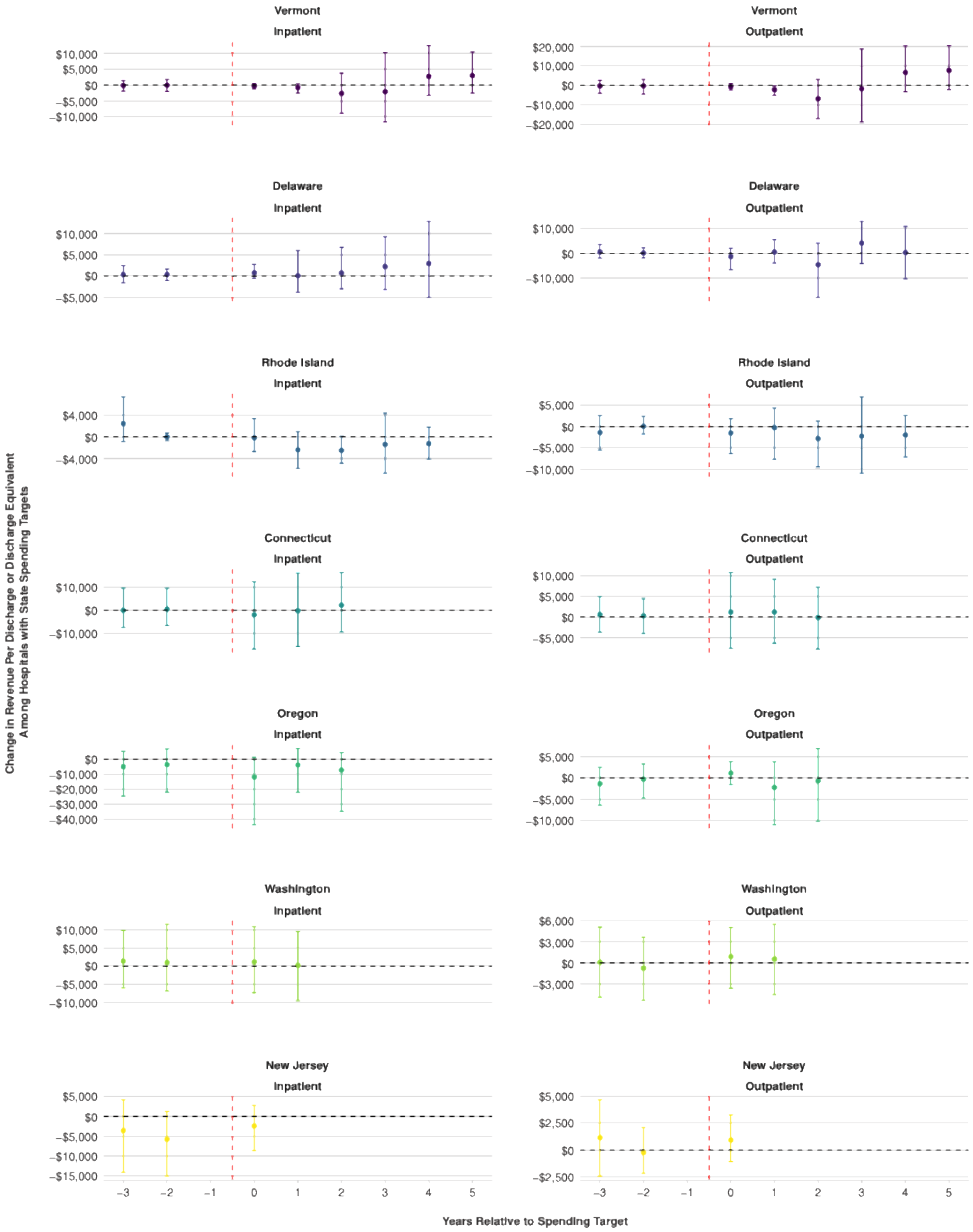

© 2026 Eibner C et al. *JAMA Network Open*.

eFigure 4. State-Specific Event-Study Plots for Hospital Price Outcomes

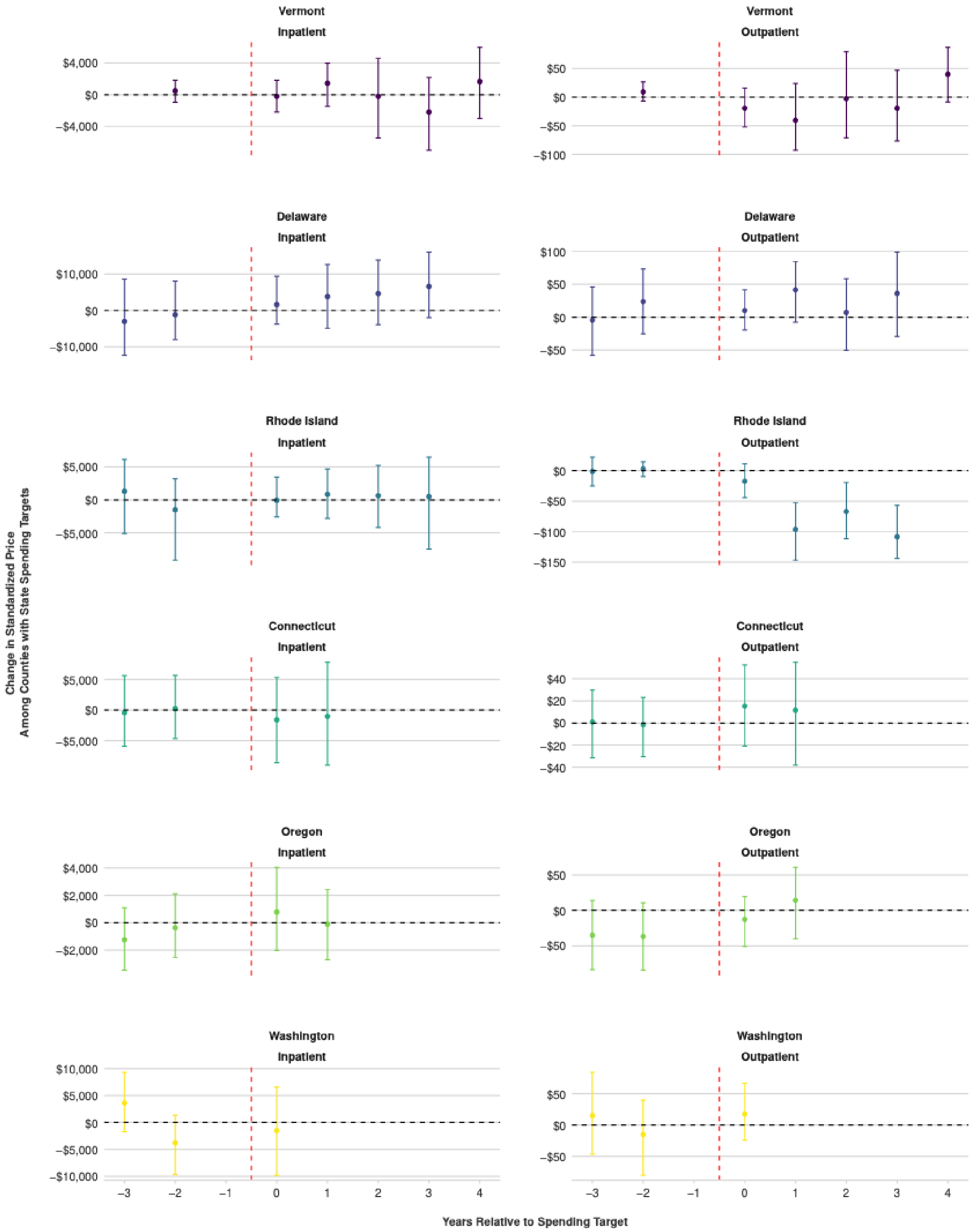

© 2026 Eibner C et al. *JAMA Network Open*.

eFigure 5. State-Specific Event-Study Plots for Premiums Outcomes

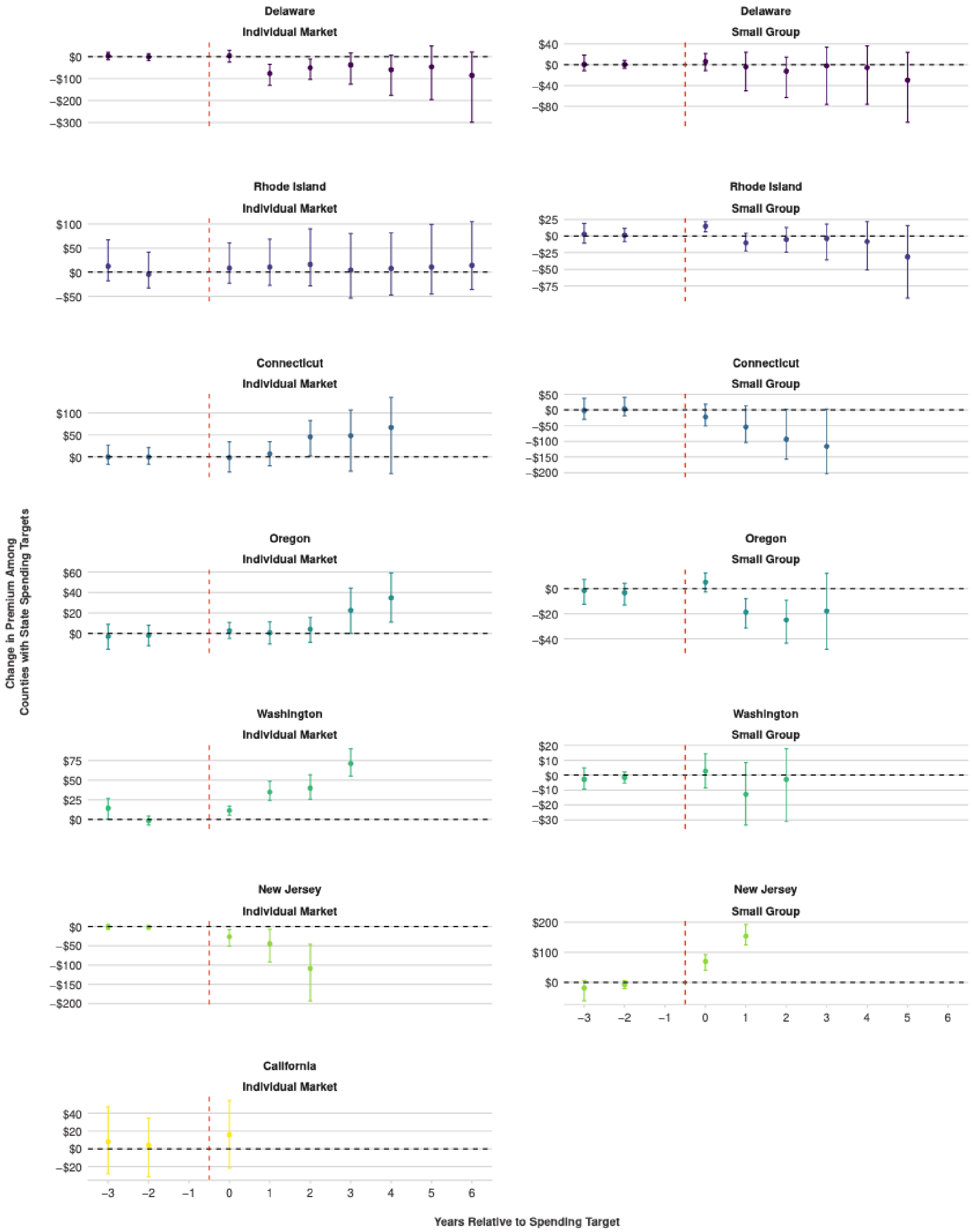

© 2026 Eibner C et al. *JAMA Network Open*.

eMethods 4. Sensitivity Analyses

To evaluate the robustness of our findings, we carried out a range of sensitivity analyses. For the hospital revenue outcomes, we conducted the following sensitivity analyses:

- Fitting the DD models without the time-varying covariates
- Fitting the DD models without the population size weighting (but still using the entropy balancing and fractionated bootstrap weights)
- Winsorizing the revenue per discharge/discharge equivalent outcomes at the 5th and 95th percentiles
- Restricting the analysis to short-term general hospitals only

For the hospital price outcomes, we conducted the following sensitivity analyses:

- Fitting the DD models without the time-varying covariates
- Fitting the DD models without the population size weighting (but still using the entropy balancing and fractionated bootstrap weights)
- Fitting the DD models without adjusting for the proportion of claims coming from an APCD

For the premiums outcomes, we conducted the following sensitivity analyses:

- Fitting the DD models without the time-varying covariates
- Fitting the DD models without the population size weighting (but still using the entropy balancing and fractionated bootstrap weights)
- Using the 2015-indexed premium, rather than the premium itself
- Including Vermont in the premiums regressions.

All of these results are presented below. None of the results depart from those presented in the paper, except the individual market premiums analysis when we include Vermont in the premiums analysis. However, as described in the paper, Vermont's usual rating rules and ongoing policy changes throughout the study period suggest that parallel trends were unlikely to have held in the post-intervention period for that state.

© 2026 Eibner C et al. *JAMA Network Open*.

eTable 7. Sensitivity Analyses of State-Specific Overall Effect Estimates for Inpatient Hospital Revenue per Discharge

State	Main	Unweighted	Winsorized	No Covariates	STGH Only
Vermont	$123 (-2,876, 4,213)	-$1,853 (-5,192, 1,754)	$17 (-3,026, 4,126)	$1,502 (-1,557, 5,379)	-$465 (-2,356, 1,696)
Delaware	$939 (-2,062, 5,426)	$1,959 (-2,076, 7,624)	$955 (-1,425, 4,304)	$1,047 (-1,508, 5,218)	$438 (-1,164, 1,812)
Rhode Island	-$2,330 (-5,235, 456)	-$381 (-3,133, 2,341)	-$2,318 (-5,287, 539)	-$2,363* (-4,664, -516)	-$2,456 (-5,384, 427)
Connecticut	-$103 (-9,589, 9,459)	-$1,559 (-9,971, 6,572)	-$336 (-4,951, 4,407)	$924 (-364, 2,060)	$351 (-3,406, 4,898)
Oregon	-$4,861 (-18,477, 1,261)	-$965 (-4,342, 1,666)	-$945 (-4,216, 1,300)	-$3,350 (-11,689, 896)	$129 (-2,127, 2,021)
Washington	-$62 (-7,473, 7,259)	$10 (-3,744, 3,999)	$43 (-2,429, 2,497)	$56 (-5,713, 5,064)	-$9 (-1,855, 1,822)
New Jersey	$688 (-1,883, 3,842)	-$600 (-5,583, 4,299)	$947 (-576, 2,907)	-$330 (-3,189, 2,514)	-$30 (-1,319, 1,359)
California	NA	NA	NA	NA	NA
Overall	-$839 (-4,276, 1,874)	-$551 (-2,573, 1,523)	$1 (-1,191, 1,137)	-$614 (-2,944, 1,322)	-$61 (-986, 925)

NOTE: * indicates $p < 0.05$.

© 2026 Eibner C et al. *JAMA Network Open*.

eTable 8. Sensitivity Analyses of State-Specific Overall Effect Estimates for Outpatient Hospital Revenue per Discharge Equivalent

State	Main	Unweighted	Winsorized	No Covariates	STGH Only
Vermont	$699 (-4,552, 7,168)	-$2,880 (-10,696, 5,655)	$400 (-4,416, 6,545)	$2,115 (-1,767, 7,371)	-$1,297 (-4,778, 2,983)
Delaware	-$680 (-6,069, 3,886)	-$3,247 (-12,649, 3,020)	$962 (-2,506, 4,814)	-$2,045 (-9,703, 3,127)	$1,584 (-575, 3,867)
Rhode Island	-$1,296 (-6,109, 1,925)	-$4,437 (-33,987, 835)	-$265 (-3,101, 2,457)	-$1,250 (-6,226, 653)	-$1,150 (-6,543, 2,118)
Connecticut	$452 (-4,551, 6,030)	-$1,018 (-6,366, 5,240)	$605 (-3,928, 5,481)	-$1,604 (-4,302, 171)	$1,019 (-4,160, 7,023)
Oregon	-$73 (-4,624, 3,448)	$184 (-3,270, 5,436)	-$515 (-5,299, 2,613)	$111 (-3,799, 3,240)	-$6 (-4,620, 3,624)
Washington	$953 (-2,358, 4,673)	-$478 (-4,287, 3,412)	$183 (-2,632, 2,704)	$669 (-1,331, 2,620)	$178 (-2,557, 2,676)
New Jersey	$635 (-1,027, 2,315)	-$294 (-2,268, 1,405)	$789 (-889, 2,498)	$1,125 (-52, 2,434)	$819 (-859, 2,585)
California	NA	NA	NA	NA	NA
Overall	$439 (-1,207, 2,126)	-$759 (-2,673, 1,237)	$248 (-1,270, 1,634)	$316 (-843, 1,411)	$276 (-1,240, 1,772)

NOTE: * indicates $p < 0.05$.

© 2026 Eibner C et al. *JAMA Network Open*.

eFigure 6. Sensitivity Analyses of Event-Study Plots for Hospital Revenue Outcomes

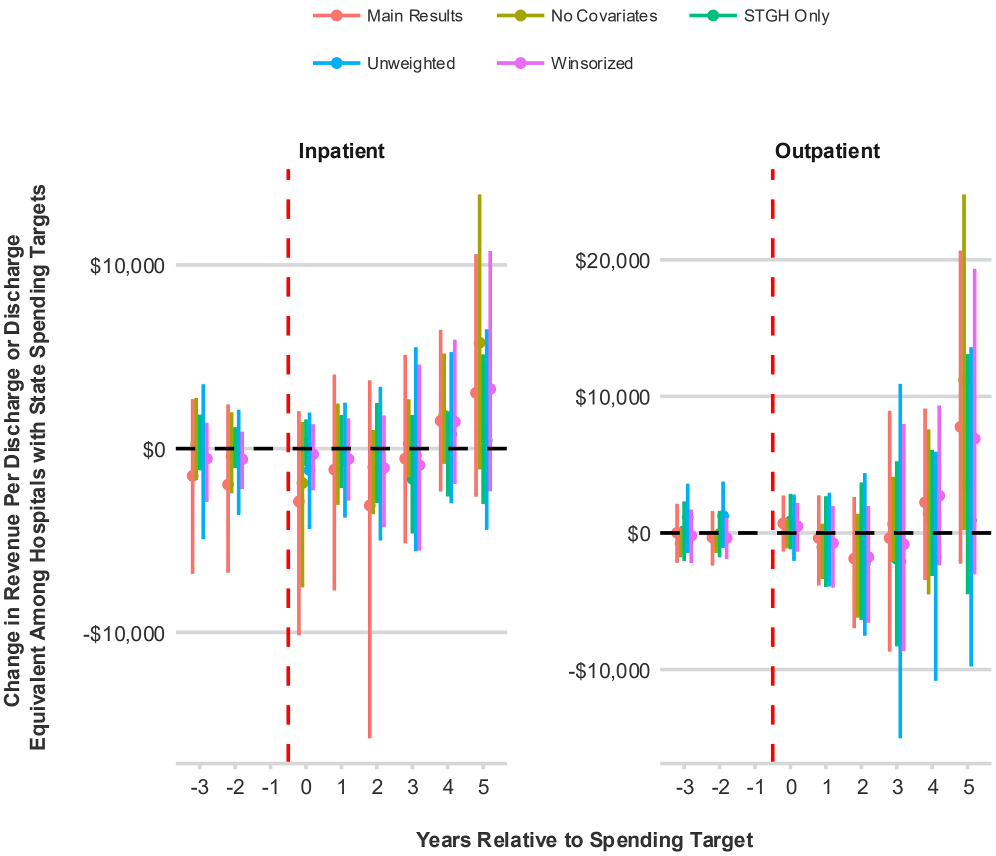

NOTE: Estimates for later years are based only on the subset of treated states with sufficient follow-up data.

© 2026 Eibner C et al. *JAMA Network Open*.

eTable 9. Sensitivity Analyses of State-Specific Overall Effect Estimates for Inpatient Hospital Price

State	Main	No APCD Adjustment	No Covariates	Unweighted
Vermont	-$7 (-2,217, 2,172)	$902 (-1,045, 2,930)	$1,421 (-1,034, 3,738)	$56 (-2,397, 2,542)
Delaware	$4,516 (-2,702, 10,068)	$4,902 (-2,367, 9,376)	$4,866 (-1,624, 9,046)	$4,789 (-1,416, 10,924)
Rhode Island	$596 (-2,211, 3,660)	$1,955 (-2,397, 6,882)	$2,739 (-2,072, 7,692)	$1,053 (-2,296, 3,996)
Connecticut	-$1,324 (-6,816, 3,838)	-$1,285 (-6,775, 3,802)	-$1,536 (-3,530, 484)	-$1,134 (-4,856, 2,606)
Oregon	$839 (-1,400, 2,964)	$952 (-1,243, 3,038)	$1,208 (-720, 2,895)	$918 (-1,008, 2,636)
Washington	-$2,057 (-9,949, 7,156)	-$2,068 (-9,966, 7,142)	-$2,677 (-9,027, 4,552)	$2,178 (-3,247, 7,981)
New Jersey	NA	NA	NA	NA
California	NA	NA	NA	NA
Overall	-$3 (-2,290, 2,417)	$285 (-2,023, 2,687)	$359 (-1,555, 2,243)	$994 (-629, 2,704)

NOTE: * indicates $p < 0.05$.

© 2026 Eibner C et al. *JAMA Network Open*.

eTable 10. Sensitivity Analyses of State-Specific Overall Effect Estimates for Outpatient Hospital Price

State	Main	No APCD Adjustment	No Covariates	Unweighted
Vermont	-$15 (-46, 22)	-$15 (-45, 20)	-$5 (-33, 23)	-$9 (-49, 37)
Delaware	$21 (-17, 58)	$21 (-18, 61)	$27 (-3, 59)	$36* (3, 70)
Rhode Island	-$55* (-88, -20)	-$93* (-154, -36)	-$102* (-177, -36)	-$50* (-75, -19)
Connecticut	$14 (-17, 43)	$13 (-18, 41)	$9 (-11, 25)	$16 (-6, 41)
Oregon	$23 (-26, 62)	$17 (-32, 57)	$30 (-11, 64)	$19 (-22, 55)
Washington	$17 (-20, 64)	$16 (-21, 64)	$18 (-8, 51)	$23 (-4, 56)
New Jersey	NA	NA	NA	NA
California	NA	NA	NA	NA
Overall	$11 (-12, 34)	$7 (-17, 31)	$12 (-7, 31)	$13 (-5, 33)

NOTE: * indicates $p < 0.05$.

© 2026 Eibner C et al. *JAMA Network Open*.

eFigure 7. Sensitivity Analyses of Event-Study Plots for Hospital Price Outcomes

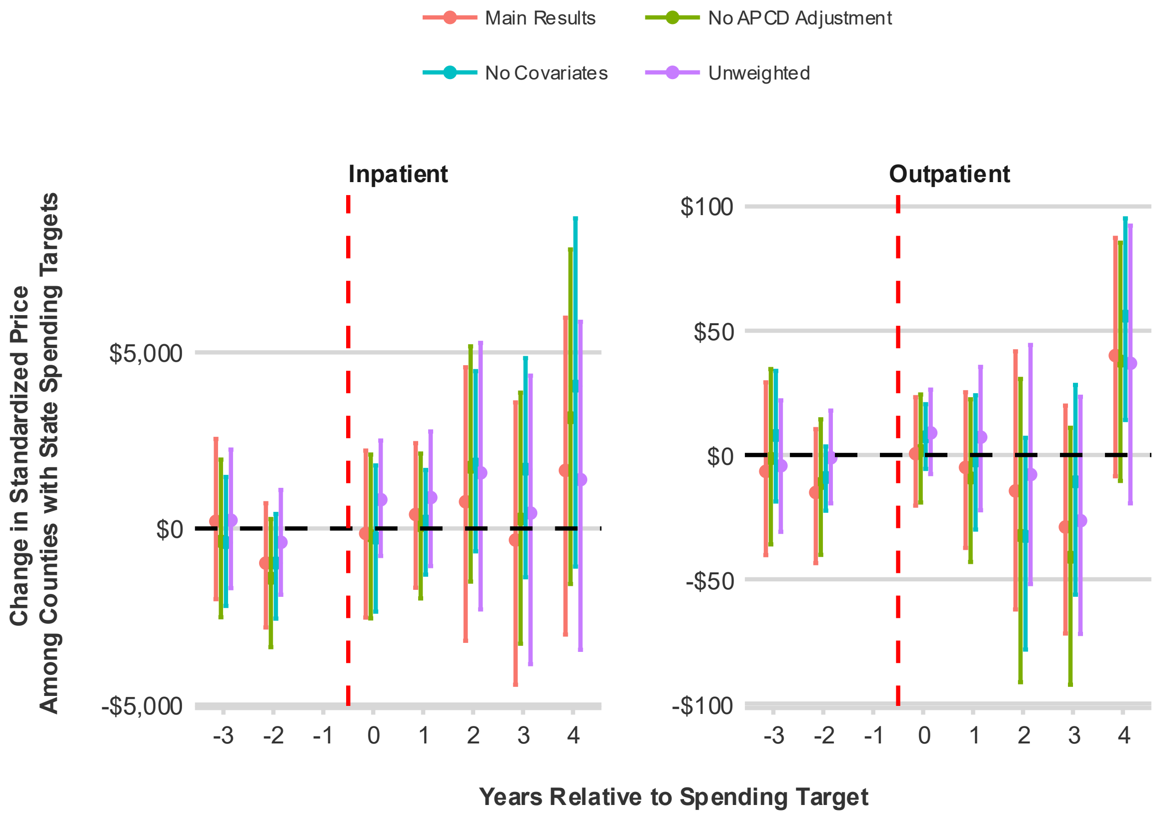

NOTE: Estimates for later years are based only on the subset of treated states with sufficient follow-up data.

© 2026 Eibner C et al. *JAMA Network Open*.

eTable 11. Sensitivity Analyses of State-Specific Overall Effect Estimates for Individual Market Premium

State	Main Results	With Vermont	No Covariates	Unweighted	Index
Vermont	NA	$76*<br>(30, 106)	NA	NA	NA
Delaware	-$49*<br>(-124, -20)	-$49*<br>(-124, -20)	-$48*<br>(-130, -15)	-$38*<br>(-96, -3)	-$11*<br>(-17, -4)
Rhode Island	$8<br>(-33, 48)	$8<br>(-33, 49)	$7<br>(-36, 51)	$9<br>(-29, 47)	$8<br>(-12, 27)
Connecticut	$32<br>(-16, 63)	$32<br>(-17, 63)	$36<br>(-8, 58)	$43*<br>(22, 64)	$12<br>(0, 22)
Oregon	$14*<br>(3, 26)	$14*<br>(3, 26)	$11*<br>(2, 20)	$12*<br>(5, 19)	$9*<br>(5, 13)
Washington	$34*<br>(22, 48)	$34*<br>(22, 49)	$33*<br>(21, 48)	$35*<br>(27, 42)	$6<br>(-7, 18)
New Jersey	-$59*<br>(-108, -22)	-$59*<br>(-108, -22)	-$60*<br>(-111, -22)	-$48*<br>(-93, -22)	-$27*<br>(-41, -11)
California	$12<br>(-28, 56)	$12<br>(-29, 56)	$11<br>(-11, 52)	$8<br>(-21, 33)	$2<br>(-11, 22)
Overall	$8<br>(-8, 26)	$14<br>(-3, 30)	$7<br>(-6, 22)	$9<br>(-4, 20)	$1<br>(-5, 9)

NOTE: * indicates $p < 0.05$.

© 2026 Eibner C et al. *JAMA Network Open*.

eTable 12. Sensitivity Analyses of State-Specific Overall Effect Estimates for Small Group Premium

State	Main Results	With Vermont	No Covariates	Unweighted	Index
Vermont	NA	-$39 (-122, 74)	NA	NA	NA
Delaware	-$5 (-47, 18)	-$4 (-46, 19)	-$10 (-54, 11)	-$12 (-56, 16)	$0 (-12, 7)
Rhode Island	-$5 (-27, 16)	-$5 (-27, 16)	-$6 (-32, 16)	-$9 (-40, 9)	-$1 (-8, 5)
Connecticut	-$72 (-112, 1)	-$72 (-113, 2)	-$65 (-102, 8)	-$43 (-89, 25)	$6 (-8, 24)
Oregon	-$12* (-26, 0)	-$13* (-27, -1)	-$14* (-27, -4)	-$15* (-24, -7)	-$4 (-10, 2)
Washington	-$3 (-20, 14)	-$3 (-20, 14)	-$3 (-19, 13)	$5 (-3, 13)	-$2 (-14, 9)
New Jersey	$117* (94, 138)	$117* (93, 137)	$112* (90, 128)	$107* (82, 128)	$32* (20, 38)
California	NA	NA	NA	NA	NA
Overall	$11 (-3, 25)	$6 (-12, 24)	$10 (-4, 24)	$13* (1, 25)	$5 (-1, 10)

NOTE: * indicates $p < 0.05$.

eFigure 8. Sensitivity Analyses of Event-Study Plots for Premium Outcomes

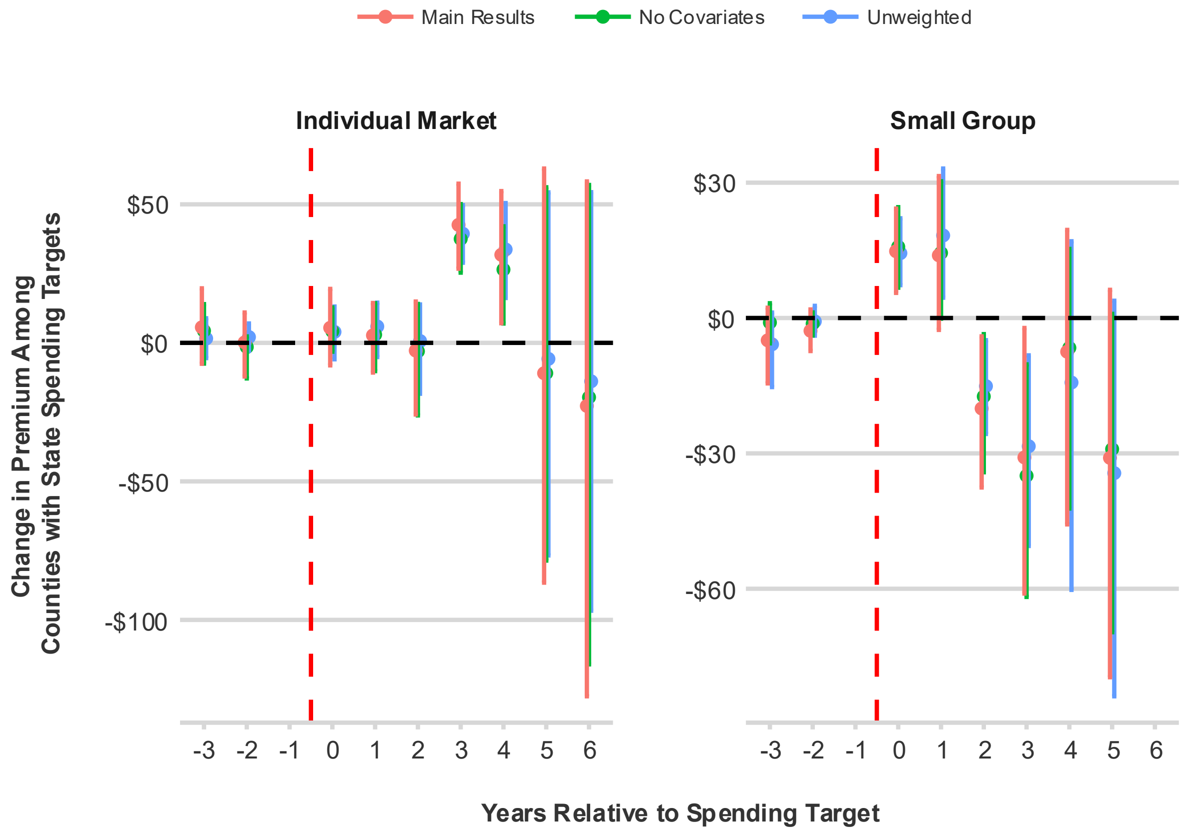

NOTE: Estimates for later years are based only on the subset of treated states with sufficient follow-up data.

© 2026 Eibner C et al. *JAMA Network Open*.

eFigure 9. Event-Study Plots for Indexed Premium Outcomes

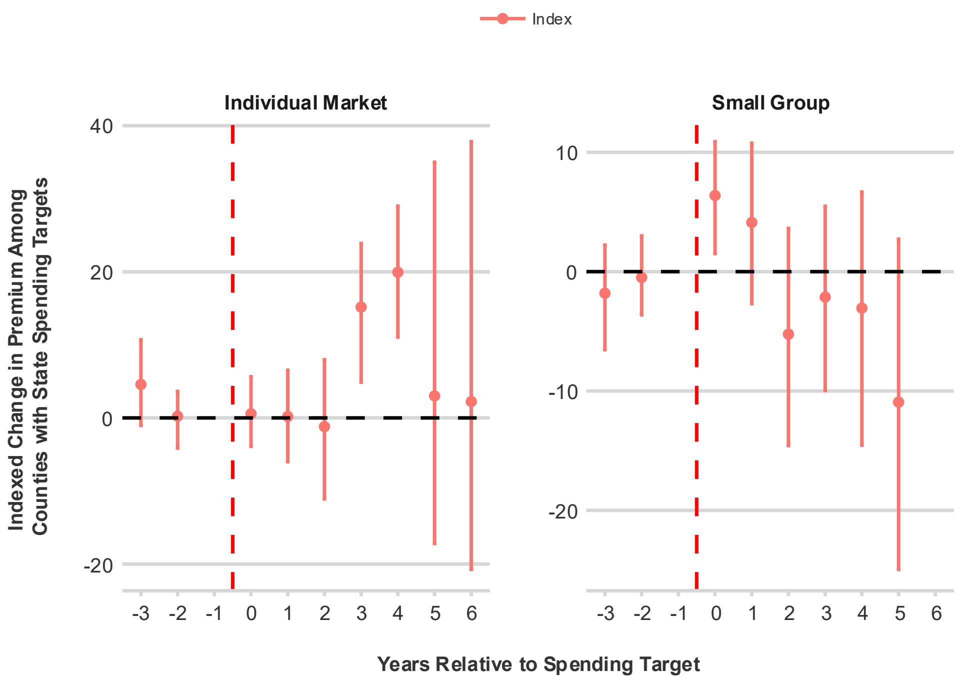

NOTE: Estimates for later years are based only on the subset of treated states with sufficient follow-up data.

© 2026 Eibner C et al. *JAMA Network Open*.

eFigure 10. Event-Study Plots for Premium Outcomes, Including Vermont

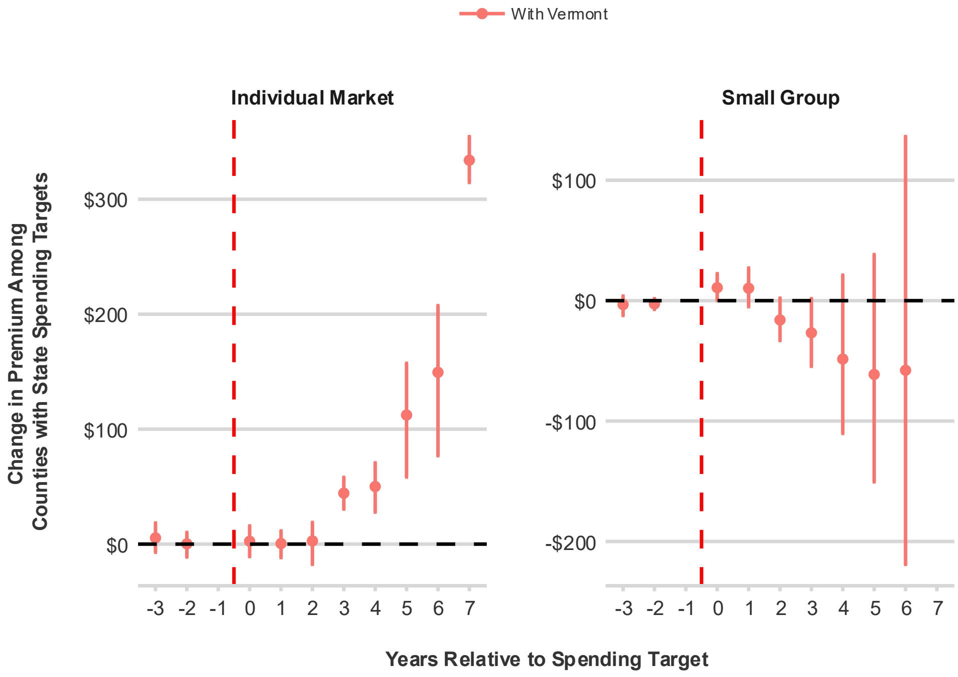

NOTE: Estimates for later years are based only on the subset of treated states with sufficient follow-up data.

© 2026 Eibner C et al. *JAMA Network Open*.

eReferences

1. Center for Health Information and Analysis. Annual report on the performance of the Massachusetts health care system. 2025.
2. Green Mountain Care Board. *Vermont all-payer ACO model total cost of care annual report performance year 5 (January – December 2022)*. 2024. https://gmcboard.vermont.gov/sites/gmcb/files/documents/ANNUAL_22TCOC%20Report_FINAL.pdf
3. State of Deleware Department of Health and Human Services. *Benchmark trend report: Calendar year 2022 results*. 2022. https://dhss.delaware.gov/wp-content/uploads/sites/4/dhcc/pdf/cy2022spendingqualitybenchmarkrpt.pdf
4. State of Rhode Island Office of The Health Insurance Commissioner. *Annual report: Health care spending and quality in Rhode Island*. 2024. https://ohic.ri.gov/sites/g/files/xkgbur736/files/2024-05/OHIC%20Cost%20Trends%20Report_20240513%20FINAL.pdf
5. Connecticut Office of Health Strategy. *Healthcare benchmark initiative Cost Growth Benchmark 2023 performance*. 2025. https://portal.ct.gov/ohs/-/media/ohs/cost-growth-benchmark/benchmark-reports-py2023/ohs-hcbi-cost-growth-benchmark-report-py2023-rev-04_24_2025.pdf
6. Oregon Health Authority. *Health care cost growth trends in Oregon, 2021-2022*. 2024. https://www.oregon.gov/oha/HPA/HP/Cost%20Growth%20Target%20documents/2024-Oregon-Cost-Growth-Target-Annual-Report.pdf
7. Health Care Cost Transparency Board. *Health care spending growth in Washington, 2019–2022*. 2024. https://www.hca.wa.gov/assets/program/data-call-report-2024.pdf
8. Governor's Office of Health Care Affordability, Transparency and the New Jersey Department of Banking and Insurance. *Health care cost growth benchmark report: Pre-benchmark year (2018–2019): Report to the New Jersey Health Care Affordability, Responsibility, and Transparency (HART) Program*. 2024. https://www.nj.gov/dobi/division_insurance/HART/reports/HealthCareCostGrowthBenchmarkReport_PreBenchmarkYear20182019.pdf
9. California Department of Health Care Access and Information. *Statewide health care spending target approval is key step towards improving health care affordability for Californians*. 2024. https://hcai.ca.gov/statewide-health-care-spending-target-approval-is-key-step-towards-improving-health-care-affordability-for-californians/
10. U.S. Census Bureau. County population by characteristics: 2010-2019. https://www.census.gov/data/tables/time-series/demo/popest/2010s-counties-detail.html
11. U.S. Census Bureau. Small Area Income and Poverty Estimates (SAIPE) Program. https://www.census.gov/programs-surveys/saipe.html
12. U.S. Bureau of Labor Statistics. County employment and wages. https://www.bls.gov/web/cewqtr.supp.toc.htm
13. Centers for Disease Control and Prevention. United States COVID-19 community levels by county. https://data.cdc.gov/Public-Health-Surveillance/United-States-COVID-19-Community-Levels-by-County/3nnm-4jni/about_data
14. U.S. Department of Agriculture Economic Research Service. Rural-urban continuum codes. 2025
15. HRSA Data Warehouse. Area health resource files. https://data.hrsa.gov/topics/health-workforce/nchwa/ahrf
16. Kane N, Berenson R, Blanchfield B, Blavin F, Arnos D, Zuckerman S. Why policymakers should use audited financial statements to assess health systems' financial health. *J Health Care Fin*. 2021;48(1)
17. Kane NM, Magnus SA. The Medicare cost report and the limits of hospital accountability: Improving financial accounting data. *J Health Polit Policy Law*. 2001;26(1
18. Rubin DB. Multiple imputation after 18+ years. *J Am Stat Assoc*. 1996;91(434):473-489.
19. Wijesuriya R, Moreno-Betancur M, Carlin JB, Lee KJ. Evaluation of approaches for multiple imputation of three-level data. *BMC Med Res Methodol*. 2020;20(1):207.
20. Austin PC, White IR, Lee DS, van Buuren S. Missing data in clinical research: a tutorial on multiple imputation. *Can J Cardiol*. 2021;37(9):1322-1331.
21. Austin PC, Giardiello D, van Buuren S. Impute-then-exclude versus exclude-then-impute: lessons when imputing a variable used both in cohort creation and as an independent variable in the analysis model. *Stat Med*. 2023;42(10):1525-1541.
22. Xu L, Gotwalt C, Hong Y, King CB, Meeker WQ. Applications of the fractional-random-weight bootstrap. *Am Stat*. 2020;74(4):345-358.

© 2026 Eibner C et al. *JAMA Network Open*.

23. Callaway B, Sant'Anna PH. Difference-in-differences with multiple time periods. *J Econom*. 2021;225(2):200-230.
24. Sun L, Abraham S. Estimating dynamic treatment effects in event studies with heterogeneous treatment effects. *J Econom*. 2021;225(2):175-199.
25. Schomaker M, Heumann C. Bootstrap inference when using multiple imputation. *Stat Med*. 2018;37(14):2252-2266.

© 2026 Eibner C et al. *JAMA Network Open*.
