# Supplementary material for: State Spending Growth Benchmarks and Hospital Revenue, Hospital Prices, and Premiums
Source: JAMA Netw Open. 2026 Feb 20;9(2):e2558283. doi: 10.1001/jamanetworkopen.2025.58283 (PMC12924095; doi:10.1001/jamanetworkopen.2025.58283)
Supplement: Supplement 3. — Data Sharing Statement [file jamanetwopen-e2558283-s003.pdf]

Data Sharing Statement

Eibner. State Spending Growth Benchmarks and Hospital Revenue, Hospital Prices, and Premiums. *JAMA Netw Open*. Published February 20, 2026.
doi:10.1001/jamanetworkopen.2025.58283

Data
Data available: Yes
Data types: Data (not involving human participants)
How to access data: eibner@rand.org
When available: With publication

Supporting Documents
Document types: None

Additional Information
Who can access the data: Data and analytic code will be made available to anyone requesting the data and/or analytic code. Please note that our study involves several data sources, and one has DUA restrictions. Data from that source will not be made available unless researchers have obtained permission.
Types of analyses: Research purposes
Mechanisms of data availability: In general without restrictions, except for the data requiring DUA approval
Any additional restrictions: The RAND HPTD requires reuse approval from individual contributors.
